# Risk Alert System to Facilitate Universal Suicide Risk Screening in Emergency Care: Protocol for a Prediction Model and Software Development Study Using a Co-Design Approach

**DOI:** 10.2196/100498

**Published:** 2026-09-16

**Authors:** Montserrat López-Fernández, Raúl Rodríguez Valderas, Ana Portillo-Van Diest, Gemma Vilagut, Miguel-Ángel Mayer, Ángela Leis, Montse Ferrer, Franco Amigo, Laura Latorre, Tatiana Leiss, Juan Manuel Ramírez-Anguita, Jorge Lemos Portela, Jordi Alonso, Manuel Pastor, Philippe Mortier

**Affiliations:** 1 Health Services Research Group Hospital del Mar Research Institute Barcelona, Catalonia Spain; 2 Department of Medicine and Life Sciences Universitat Pompeu Fabra Barcelona, Catalonia Spain; 3 Institut de Salut Mental Hospital del Mar Barcelona, Catalonia Spain; 4 CIBER de Epidemiología y Salud Pública, Instituto de Salud Carlos III (CIBERESP, ISCIII) Madrid, Madrid Spain; 5 Research Programme on Biomedical Informatics (GRIB) Hospital del Mar Research Institute Barcelona, Catalonia Spain; 6 Data Science Unit Hospital del Mar Barcelona, Catalonia Spain; 7 Department of Population Health Sciences Duke University Durham, NC United States

**Keywords:** artificial intelligence, clinical decision support systems, electronic health records, emergency service, hospital, machine learning, mass screening, risk assessment, self-injurious behavior, stakeholder participation, suicide

## Abstract

**Background:**

Suicide is a leading cause of preventable mortality and a major contributor to years of life lost. Many people who later die by suicide present to emergency departments in the months before death, often for reasons not explicitly related to self-harm and without receiving a risk assessment. Universal suicide risk screening in emergency care can improve detection but is difficult to implement because of time constraints, workflow disruption, and limited capacity to respond to increased identification.

**Objective:**

The Clinical Risk Alert System for Suicide Risk Screening in Emergency Settings (CARES) project aims to lower the practical threshold for universal screening by developing a risk alert system software prototype for use among patients presenting to emergency care, prompting a brief, validated suicide risk screening and clinician-led assessment.

**Methods:**

CARES will develop machine learning–based prediction models for subsequent nonlethal intentional self-harm and suicide within 1, 6, and 12 months after discharge from an index emergency department visit, using linked, population-based electronic registries from Catalonia (Spain), including electronic health records, mortality data, administrative sociodemographic variables, and a specific self-harm register. Index visits will include emergency department contacts in individuals aged 6 years or older without self-harm–related chief complaints, with predictors defined from information recorded in the prior 12 months. Models will be trained with approaches addressing rare outcomes and unequal sampling probabilities, and evaluated using an independent test set and temporal validation. The risk alert system software prototype will be designed as a web-based application with a backend hosting trained models and a frontend that allows data entry and displays risk as descriptive text and visualizations in absolute and relative terms compared with same-age and same-sex peers. Implementation research will use a co-design process with a user advisory group (UAG; clinicians, people with lived experience, caregivers, and stakeholder organizations), guided by the Medical Research Council framework for complex interventions, to define alert thresholds, response pathways, usability requirements, and training needs.

**Results:**

The CARES project was funded in March 2023. The project uses linked electronic registry data from Catalonia covering 2014-2019, including 2,982,736 eligible emergency department index visits from 603,098 patients. The first UAG meeting was held in December 2024. As of May 2026, the project is developing the initial prediction models and software backend, while iteratively refining the frontend through UAG meetings. Data analysis is ongoing, and the main results are expected to be published in spring 2027.

**Conclusions:**

CARES will deliver an experimental proof-of-concept risk alert system designed to support future targeted suicide risk assessment within emergency workflows while keeping clinical decision-making with clinicians and patients. If feasible and acceptable, this approach could improve detection of otherwise unrecognized risk and inform future real-time integration and evaluation within routine emergency care.

**International Registered Report Identifier (IRRID):**

DERR1-10.2196/100498

## Introduction

Suicide is a significant yet preventable public health issue, accounting for over 700,000 deaths annually and an estimated loss of 34.6 million years of life [1,2]. The most recent data (2024) indicate that 3953 people died by suicide in Spain (2902 male and 1051 female individuals), including 547 in Catalonia (400 male and 147 female individuals). These figures correspond to suicide mortality rates of approximately 4 per 100,000 in women and 12 per 100,000 in men [3]. Suicide is the leading cause of death among individuals aged 20-24 years in Spain, and each suicide is estimated to affect or bereave up to 135 individuals [4]. As many suicides go undetected or unregistered, the true extent and scale of the impact of suicide remains largely unknown [2,5].

Emergency departments (EDs) are increasingly recognized as key settings for suicide risk detection within the general population [6-9]. Empirical evidence suggests that a significant proportion (between 38.8% and 59.9%) of individuals who die by suicide had presented to an ED in the months or the year preceding their death, yet were not screened for suicide risk during these encounters [10-14]. Suicidal thoughts are often not recognized without the implementation of standardized assessments. Estimates suggest that between 4.6% and 11.6% of adult patients presenting to the ED for nonpsychiatric reasons report suicidal ideation when actively screened [15-17]; at pediatric EDs this is 3.6%-5.7% [18-21]. Detected patients also included patients explicitly reporting planning suicide or having recent suicide attempts. See Multimedia Appendix 1 for a full literature review on the ED as a key setting for suicide risk detection.

Implementing universal suicide risk screening protocols in EDs may improve detection of individuals at risk and create opportunities for timely intervention [7,22-25]. Timely identification of individuals at risk of nonlethal intentional self-harm (NLISH) and suicide is essential to reducing suicide mortality [26]. The effectiveness of universal suicide risk screening in EDs could be enhanced through the integration of machine learning–based predictive models that automatically analyze patients’ electronic health records (EHRs) [27-32]. In this paper, we present CARES, a research project aimed at developing a prototype of a suicide risk alert system (RAS) software, conceived as a predictive clinical decision support system. This system leverages machine learning–based prediction models trained on patients’ registry data to enable risk stratification for NLISH and suicide among individuals presenting to EDs for complaints other than self-injurious thoughts or behaviors. The RAS aims to generate alerts for clinicians, prompting a targeted assessment using a brief, validated suicide risk screening tool. In doing so, the system aims to lower the threshold for implementing universal suicide risk screening by increasing its efficiency, enabling targeted assessment of a high-risk subgroup instead of systematic screening of all ED patients. The RAS will be cocreated with end users in order to increase its acceptability and feasibility, and to maximize future implementation within the health care system of Catalonia, Spain. The involvement of stakeholders in designing clinical interventions has gained increasing recognition as a means of bridging the knowledge-action gap. Cocreation, an umbrella concept encompassing co-design (collaborative planning) and coproduction (stakeholder involvement in implementation), has emerged as a participatory approach that enhances innovation, facilitates implementation, increases initiative success, and strengthens equity in health initiatives [33-35]. In suicide prevention, this approach is particularly relevant given the complexity and sensitivity of care contexts. Many individuals presenting with emotional distress, especially in emergency settings, report negative care experiences, underscoring the need for services that meaningfully incorporate the perspectives of both patients and providers. Cocreated interventions in suicide prevention have shown promise in addressing these challenges, and recent reviews highlight growing applications of co-design in self-harm interventions, including those implemented in EDs [36]. See Multimedia Appendix 1 for a full literature review on cocreation in suicide interventions.

This protocol paper briefly summarizes the rationale for CARES, presents its aims and methods, and reports findings from the first stakeholder meeting.

## Methods

### Aims

CARES integrates expertise in clinical mental health, suicide prevention, public health, biostatistics, and biomedical informatics to develop a RAS prototype (Figure 1) capable of generating risk alerts for self-harm and suicide among patients presenting to EDs with chief complaints not directly related to self-injurious thoughts or behaviors (ie, nonpsychiatric chief complaints or psychiatric chief complaints but without self-injurious thoughts or behaviors). These alerts will be triggered by machine learning prediction models developed using centralized electronic registry data, including routinely collected health care, administrative, mortality, and case-level data from a dedicated self-harm register linked to a regional suicide prevention surveillance program. The alerts are intended to identify patients who may benefit from an in-person targeted assessment using a brief, validated risk screening tool. The RAS therefore seeks to automate the initial step of universal risk screening, addressing time constraints in ED settings, by allocating valuable resources to those most in need for targeted in-person risk assessments. The project prioritizes a co-design process with all key stakeholders, including clinicians and individuals with lived experience, to explore the development of a RAS-based intervention for universal suicide risk screening (Figure 1 [37]). This participatory approach aims to improve the integration of the CARES tool into clinical practice by identifying barriers, enhancing usability, and increasing acceptance among health care professionals and patients. Ultimately, the RAS prototype will serve as a proof of concept to support future research on its effectiveness in reducing self-harm and suicide risk, and to inform its progressive real-time integration into clinical workflows, enabling universal suicide risk screening in emergency care settings.

**Figure 1:**
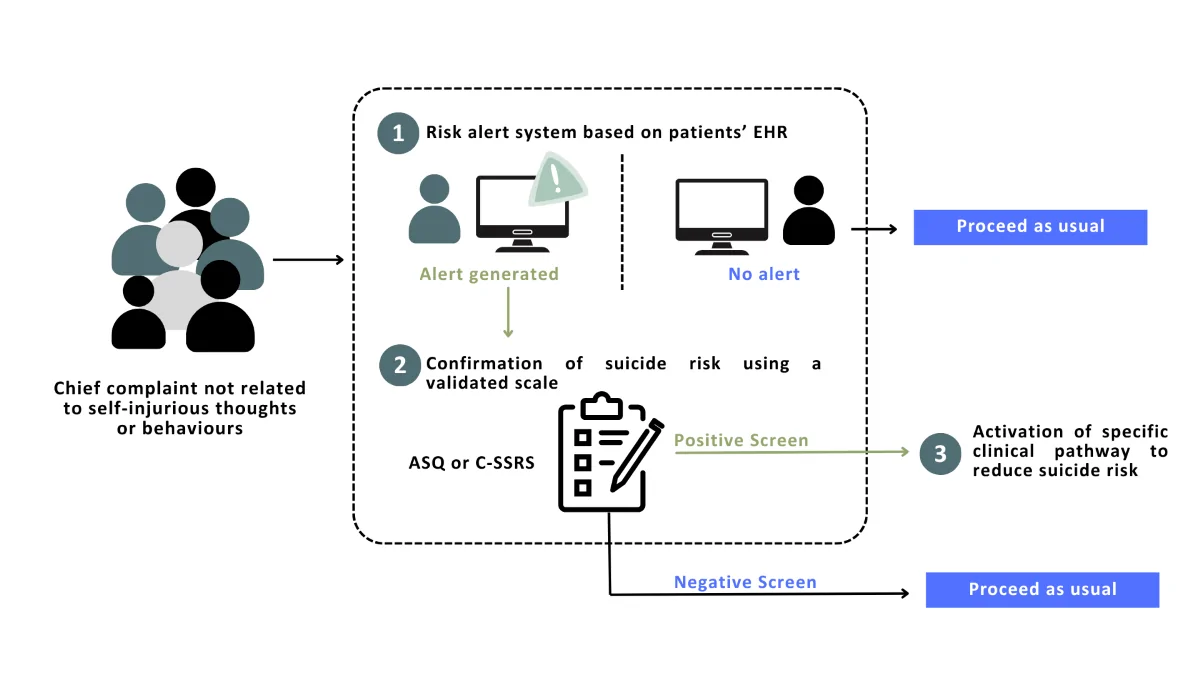
Universal suicide risk screening with electronic health record (EHR)–based automatic suicide risk alerts as proposed in the Clinical Risk Alert System for Suicide Risk Screening in Emergency Settings project. Patients whose chief complaint is not directly related to self-injurious thoughts or behaviors are screened using a validated tool only if an alert is triggered based on automatic analysis of patients’ EHRs using a risk alert system based on machine learning–based risk prediction models. Patients who screen positive are then directed through a specific clinical pathway designed to reduce suicide risk. ASQ: Ask Suicide-Screening Questions; C-SSRS: Columbia–Suicide Severity Rating Scale.

The CARES project integrates 3 interrelated methodological components: the development of machine learning–based prediction models for self-harm and suicide using electronic registry data; the design and development of the RAS software; and implementation research aimed at advancing the tool to technology readiness level 4, defined as an experimental proof of concept validated in artificial environments, but not yet tested in real-time clinical settings. Accordingly, CARES will not evaluate whether the RAS improves suicide risk detection, clinical decision-making, clinician behavior, or patient outcomes in real ED settings; these questions will be addressed in subsequent feasibility, implementation, and effectiveness studies.

### Development of Machine Learning–Based Prediction Models

#### Available Electronic Registry Data

The data used for the development of machine learning–based prediction models for intentional self-harm and suicide consists of individual-level quantitative structured data extracted from existing electronic registries from Catalonia, Spain. Electronic registry data were provided by the Agency for Health Quality and Assessment of Catalonia, which oversees the Public Data Analysis for Health Research and Innovation Programme [38,39]. The personal health care identification number assigned to all residents in Catalonia was used for accurate linkage of information between the registers.

Mortality data came from Spain’s National Statistics Institute (INE), covering the period 2014 to 2019, and included information on death by suicide, including date of death. Determination of cause of death (categorized into suicide, external causes other than suicide, and natural causes) was ascertained using *International Statistical Classification of Diseases, Tenth Revision* (*ICD-10*) codes, aligning with World Health Organization (WHO) criteria, and relies on medical death certificates and statistical death registers (natural deaths) and judicial statistical death registers (unnatural deaths). The INE follows Eurostat’s European Statistics Code of Practice for data quality assurance.

Routinely collected EHR data representing the entire Catalan public health care system came from the Catalan Health Service and includes information on ED visits (2014-2019), psychiatric hospitalizations (2008-2019), outpatient mental health care visits (2008-2019), general hospitalizations (2007-2019), and primary care visits (2010-2019). Diagnoses included in EHR data used the *International Classification of Diseases, Ninth Revision, Clinical Modification* (*ICD-9-CM*); *ICD-10*; and *International Classification of Diseases, Tenth Revision, Clinical Modification* (*ICD-10-CM*) classification systems. Catalonia’s health care system provides universal coverage funded through taxation [40]. The registries are used for health care quality monitoring and reimbursement, with data quality ensured through verification and systematic checks by health documentalists in the Catalan Health Department [41].

Data on clinically confirmed self-harm episodes came from the Catalonia Suicide Risk Code (CSRC) program, an integrated suicide-prevention protocol within the Catalan public health care system (2014-2019) with complete population coverage. This protocol mandates face-to-face psychiatric evaluations for Catalan residents presenting with self-harm or suicide risk in any public health care setting.

Administrative data came from the Catalan Health Service’s central population register and included sex, age, socioeconomic group, country of origin, and income level.

#### Sample

Using the Catalan Health Service’s central population register, we identified all residents of Catalonia (Spain) who were alive at any point between January 1, 2014, and December 31, 2019 (n=8,662,155). A random stratified sampling design disproportionately sampling for outcome and exposure status (enrichment), similar in principle to a case-cohort design [42,43], was applied to enhance computational efficiency in the analysis of rare outcomes and exposures while preserving population representativeness. We selected all individuals with recorded suicidal ideation or self-harm events and a random subsample of noncases. Among noncases, we oversampled individuals with indicators of mental disorder history available in the central population register using disproportionate stratified random sampling. Inverse selection probability weights corrected for unequal selection probabilities, enabling the development of unbiased prediction models that are generalizable to the original target population. A detailed description of the sampling and weighting procedure is provided in Multimedia Appendix 2.

From this dataset, we will select all ED visits not related to self-injurious thoughts or behaviors with age ≥6 years at discharge. These visits will serve as index visits, marking time zero for prediction and defining the starting point from which the 1-month, 6-month, and 12-month prediction windows (event horizons) will be evaluated. The analytic dataset will include 2,982,736 index visits corresponding to 603,098 patients during the period 2014-2019. For individuals with multiple eligible ED visits, all visits will be included provided that they were separated by a sufficient temporal interval.

#### Outcome Variables

The adverse clinical outcomes predicted by the models are NLISH and suicide occurring after discharge from the index ED visit. Separate prediction models will be developed for 1-month, 6-month, and 12-month event horizons. The 1-month horizon is intended to provide shorter-term risk estimates that may be more closely aligned with ED decision-making and immediate follow-up planning, whereas the 6- and 12-month horizons will capture medium- and longer-term risk relevant to continuity of care and care pathway planning. To address undercoding of NLISH, NLISH was defined using 2 methods. First, health care visits for self-harm were identified using a 3-step hierarchical approach. A first step identified all ED contacts for intentional self-harm using standard *ICD-10* codes X60*-X84*. Next, ED contacts for undetermined self-harm were identified using standard *ICD-10* codes Y10*-Y32*. In a third step, we identified ED contacts for probable self-harm using *International Classification of Diseases* (*ICD*) codes based on a literature search [44]. *ICD-10* codes used in this third step included S51.8-S51.9, S55.0-S55.1, S61.8-S61.9, S65.0-S65.1, T39*, T40.0-4, T40.6, T42.3-T42.4, T42.6-T42.7, T43*, and T50.7 [45-48]. See Table 1 for corresponding *ICD-9-CM* and *ICD-10-CM* codes used in the 3 steps described previously. Second, episodes with a clinically confirmed method of self-harm in CSRC program data. The first event of NLISH (registered in one or both of the data sources) was used to code the outcome event. Suicide was defined as death resulting from intentional self-harm (*ICD-10* codes, X60-X84).

**Table 1:** International Classification of Diseases codes to identify self-harm in routine electronic health care data. Asterisks “*” indicate inclusion of all subcodes.a

	ICD-9-CM^b^	ICD-10-CM^c^	ICD-10^d^
Intentional self-harm	E950*-E958*: Self-poisoning, hanging, strangulation, suffocation, firearms, jumping, others	T14.91*: Suicide attempt T36*-T50* with a 5/6th character of 2: Drug poisoning (overdose) T51*-T64*, T65.0*-1*, T65.3*-9* with a 5/6th character of 2: Toxic effects of nonmedicinal substances T71* with a 5/6th character of 2: Asphyxiation, suffocation, strangulation X71*-X83*: Drowning and submersion; firearms; explosive or thermal material; sharp or blunt objects; jumping from a high place; jumping or lying in front of a moving object; crashing of a motor vehicle; others	X60*-X64*: Drug poisoning (overdose) X65*-X69*: Toxic effects of nonmedicinal substances X70*: Asphyxiation, suffocation and strangulation X71*-X84*: Drowning and submersion; firearms; explosive or thermal material; sharp or blunt objects; jumping from a high place; jumping or lying in front of a moving object; crashing of a motor vehicle, others
Undetermined self-harm	E980*-E987*, E988.1-8: Self-poisoning, hanging, strangulation, suffocation, firearms, jumping, others	T36*-T50* with a 5/6th character of 4: Drug poisoning (overdose) T51*-T64*, T65.0*-1*, T65.3*-9* with a 5/6th character of 4: Toxic effects of nonmedicinal substances T71* with a 5/6th character of 4: Asphyxiation, suffocation, strangulation Y21*-Y32*: Drowning and submersion; firearms; explosive or thermal material; sharp or blunt objects; jumping from a high place; jumping or lying in front of a moving object; crashing of a motor vehicle; others	Y10*-Y14*: Drug poisoning (overdose) Y15*-Y19*: Toxic effects of nonmedicinal substances Y20*: Asphyxiation, suffocation, strangulation Y21*-Y32*: Drowning and submersion; firearms; explosive or thermal material; sharp or blunt objects; jumping from a high place; jumping or lying in front of a moving object; crashing of a motor vehicle; others
Injuries and intoxications possibly related to self-harm	881*; 903.2-903.4 and no external cause code is registered: Open wound of elbow, forearm, and wrist; injury radial/ulnar vessels; injury palmar artery 965*; 967*; 969* and no external cause code is registered: Poisoning by analgesics, antipyretics, antirheumatics, sedatives and hypnotics 994.7 and no external cause code is registered: Asphyxiation/strangulation	S51.00*; S51.80*; S55.0*-S55.1*; S61.5*; S65.0*; S65.1*: Open wound of elbow/forearm/wrist, injury of ulnar/radial artery at forearm/wrist/arm level T36*-T50* with 5/6th character missing: Drug poisoning (overdose) T51*-T64*, T65.0*-1*, T65.3*-9* with 5/6th character missing: Toxic effects of nonmedicinal substances T71* with 5/6th character missing: Asphyxiation, suffocation, strangulation	S51.8-S51.9, S55.0-S55.1, S61.8-S61.9, S65.0-S65.1: Open wound of elbow/forearm/wrist, injury of ulnar/radial artery at forearm/wrist/arm level T39*, T40.0-4, T40.6, T42.3-T42.4, T42.6-T42.7, T43*, T50.7: Drug poisoning (overdose), including nonopioid analgesics and antipyretics, opioids and other narcotics, antiepileptics and sedative-hypnotics, antidepressants, antipsychotics, psychostimulants, other psychotropic drugs, and related substances

^a^Selection of International Classification of Diseases (ICD) codes for the “Injuries and intoxications possibly related to self-harm” category was based on a literature search. For selected International Classification of Diseases, Ninth Revision, Clinical Modification (ICD-9-CM) injury and poisoning codes (881*; 903.2-903.4; 965*; 967*; 969*; and 994.7), classification of intent required an accompanying external cause (E-) code indicating intentional self-harm, undetermined intent, or accident. When no E-code was recorded, intent could not be ascertained; these records were therefore classified as injuries and intoxications possibly related to self-harm. For International Classification of Diseases, Tenth Revision, Clinical Modification (ICD-10-CM) poisoning and injury codes (S- and T-codes), the fifth or sixth character was used to distinguish intentional self-harm, undetermined intent, or absence of specified intent. Records in which this character was missing or did not specify intent were classified as injuries and intoxications possibly related to self-harm. For ICD-10-CM codes, where applicable, only initial encounters were considered, defined by ICD-10-CM injury and poisoning codes carrying a seventh-character extension of “A,” with subsequent encounters (“D”) and sequelae (“S”) excluded. International Classification of Diseases, Tenth Revision (ICD-10) codes were used only in <1% of cases and correspond to legacy World Health Organization ICD-10 coding present in a small subset of registry records.

^b^ICD-9-CM: International Classification of Diseases, Ninth Revision, Clinical Modification.

^c^ICD-10-CM: International Classification of Diseases, Tenth Revision, Clinical Modification.

^d^ICD-10: International Classification of Diseases, Tenth Revision.

#### Predictor Variables

The features used for the prediction models (predictor variables) reflect the patients’ available clinical information in the 12 months prior to the index ED visit. These will include sociodemographic characteristics; prior self-harm and suicidal ideation; mental and substance use disorders; nonpsychiatric (somatic) clinical conditions; psychotropic medication dispensing; health care use patterns; and indicators of psychiatric and nonpsychiatric inpatient and outpatient care. Diagnostic groupings will be defined at a clinically interpretable category level rather than at the individual *ICD* code level. *ICD*-based diagnostic predictors will be constructed using multiple established frameworks. Mental and substance use disorder predictors will be defined according to the WHO *ICD-10* Classification of Mental and Behavioral Disorders and corresponding official conversion tables [49]. For both psychiatric and nonpsychiatric clinical conditions, diagnostic codes will also be aggregated using the European shortlist of causes of death [50], the Clinical Classifications Software grouping system [51], and prior registry-based feature construction approaches (eg, [52]). Composite predictor variables reflecting mental and/or somatic comorbidity will also be created. Psychotropic medication exposure will be defined by grouping Anatomical Therapeutic Chemical classification codes at the pharmacological subgroup level.

#### Prediction Model Development

We will develop and validate a series of clinically interpretable prediction models which will enable the accurate stratification of patients according to risk for the key adverse clinical outcomes. We will develop machine learning–based models, specifically random forest models. Prior to model development, feature selection will be performed by removing predictors with very low prevalence in the dataset or very low association with the outcome. To address class imbalance, algorithmic class weighting (using balanced class weights) will be applied during model training to account for the imbalance between outcome-positive and outcome-negative observations. Model hyperparameters will be optimized using a grid search approach. Temporal validation will be performed by evaluating model performance on data from later time periods than those used for model development. Concretely, the dataset will be split temporally at the visit level, with the most recent 20% of observations reserved as an independent temporal test set that will not be used during model training, feature selection, hyperparameter tuning, calibration, or threshold selection. Repeated ED visits from the same patient will be retained as separate index visits when they meet the predefined temporal separation criteria. The primary validation strategy will use a temporal split at the visit level; therefore, the same patient may contribute earlier visits to model development and later visits to testing. This was chosen to mimic a realistic deployment scenario in which future predictions may be generated for patients whose historical data contributed to model development. As a sensitivity analysis, we will compare model performance with a stricter patient-level approach retaining 1 randomly selected eligible visit per patient.

To prevent data leakage, predictors for each index ED visit will be constructed exclusively from information recorded before or at discharge from that visit, using the predefined look-back window. No information recorded after the index visit, including subsequent health care contacts, diagnoses, medication dispensing, CSRC entries, self-harm events, or mortality data during the follow-up period, will be used as model input. Feature selection, hyperparameter tuning, probability calibration, and alert-threshold selection will be performed using only the training and validation data; the independent temporal test set will remain untouched until final model evaluation. Temporal validation will further ensure that model performance is assessed on visits occurring after the model-development period.

Missing data will be handled according to the type of predictor. For registry-based event variables, including diagnoses, medication dispensing, prior self-harm, and health care use, absence of a recorded code or event during the look-back period will be coded as absence of that recorded event. These variables will therefore not be imputed, because nonrecorded clinical events reflect limitations of routine data capture rather than classical missing values. For administrative or sociodemographic variables with genuinely missing values, missingness will be described and handled using predefined missing categories or missingness indicators. The possibility of incomplete recording, particularly for psychiatric diagnoses and self-harm events, will be acknowledged as a limitation when interpreting model performance.

Model development, calibration, and performance evaluation will be conducted separately for each outcome and prediction horizon. Model performance will be evaluated using discrimination, calibration, and clinically relevant threshold-based metrics. Discrimination will be assessed using the area under the receiver operating characteristic curve and the area under the precision-recall curve, the latter being particularly informative for rare outcomes. Overall classification performance will also be summarized using accuracy and the Matthews correlation coefficient, which provides a balanced measure of performance in imbalanced datasets. For clinically relevant alert thresholds, we will estimate sensitivity, specificity, positive predictive value (PPV), and the proportion of ED visits that would trigger an alert, the latter serving as an indicator of potential workload burden and alert fatigue. We will examine threshold-based performance under clinically meaningful operating points, including specificity and PPV at fixed sensitivity levels (eg, sensitivity=0.70), and sensitivity and PPV at fixed specificity levels (eg, specificity=0.95). This will allow us to compare scenarios prioritizing broad case detection with scenarios prioritizing fewer false-positive alerts and lower clinical workload. Calibration will be assessed by comparing predicted and observed risks across risk strata, using calibration plots and summary measures such as the calibration intercept, calibration slope, and Brier score. Final model and threshold selection will consider discrimination, calibration, interpretability, PPV, expected workload burden, and context-specific trade-offs between sensitivity and specificity.

To enable clinical interpretability of prediction models [53], a set of most important predictors will be identified for each model and local explanations will be obtained using Local Interpretable Model-Agnostic Explanations to facilitate interpretation of individual predictions.

Potential prediction bias in the prediction models with regard to relevant variables (eg, sex, age group, and socioeconomic status) will be investigated and, where possible, mitigated by systematically including these variables as fixed covariates throughout model development as well as by developing separate models stratified by different values of these variables, whenever it will be considered necessary.

### RAS Prototype Software Development

The RAS software is a classical web application, with a separate backend and frontend. Development of the RAS software backend will consist of implementing the machine learning–based prediction models and maintaining a repository of trained prediction models that can be applied to new patient visits. Using this system, and based on the data of an individual patient, the system will assign a predicted risk score for the key adverse clinical outcomes. The software will work as a web service, exposing a Representational State Transfer application programming interface through which it will interchange input and output data with the frontend.

Development of the software frontend will consist of designing a web-accessible graphical user interface (GUI) that facilitates intuitive and user-friendly human-system interactions. The RAS will enable clinicians to receive automatic alerts for potential suicide risk according to EHR data and the next steps for complete risk assessment. The frontend will provide a form for entering patient data, which will be transmitted to the backend to generate risk predictions. Predicted risk will be presented as descriptive text and graphical visualizations expressing the patient’s risk in both absolute and relative terms, compared with the distribution of risk among same-age and same-sex peers presenting with ED visits not related to self-injurious thoughts or behaviors. It is important to stress that final risk assessment will not be entirely data-driven but will also be complemented with a validated scale (eg Ask Suicide-Screening Questions [ASQ] or Columbia–Suicide Severity Rating Scale [C-SSRS]), and when required, through a complete assessment based on expert opinion. Risk for intrusive interventions provoked by false-positive model predictions will be prevented by the fact that the final decision regarding treatment and individual rights restriction always lies with the end user (clinician and/or patient) and never the RAS. In future clinical testing, any alert generated by the RAS would therefore be considered a prompt for further assessment rather than a diagnostic classification or treatment recommendation. The appropriate downstream pathway, including escalation procedures and documentation requirements, will be defined through the co-design process before any real-time clinical deployment.

Evaluation of software development will consist of both technical validation (no bugs and adherence to specifications) and functional validation (verifying whether the software meets all users’ real needs). Importantly, we aim for the personalized RAS to be used as a stand-alone application, not depending on the connection with electronic health care systems for its functioning. This means that the input data can consist entirely of user-entered data, that is, clinicians providing all necessary input data manually through the software GUI. Although this will add user burden at this stage of RAS development, this is necessary to provide a feasible and flexible software that can also be tested and fine-tuned in health care settings with poor system integration [47]. To lower user burden in terms of data that needs to be entered manually, we will test, in a pilot study, the use of prediction models based on sets of predictors with highest predictive accuracy (and minimal loss of overall model prediction accuracy), and we will explore the implementation of multistage assessments, first prioritizing models with high sensitivity, and subsequently using models with optimum sensitivity and specificity trade-off.

### Cocreation of Universal Suicide Risk Screening in the ED

Cocreation is understood as a two-phase process: (1) co-design, which focuses on the development of solutions, and (2) coproduction, which refers to the implementation of the agreed-upon intervention [34]. Within the scope of the CARES project, our implementation research will initially focus on the preliminary identification of core implementation elements and the co-design of the RAS tool. This participatory approach integrates real-world knowledge and promotes local stakeholder ownership and buy-in, which is essential for the future adoption and sustainability of universal suicide risk screening.

The project has established a user advisory group (UAG) that brings together individuals with lived experience and their informal carers, frontline clinicians from general and psychiatric emergency services from all 3 types of emergency services in Catalonia (including hospital emergency services, primary care emergency centers, and the medical emergency system), and suicide prevention associations. Participants were recruited by personal invitations through their clinical settings or suicide prevention associations, and through snowball sampling. The UAG meetings will be moderated by the research team and primarily be held in person, with hybrid or online formats available as needed.

A specific objective of the co-design process will be to develop a preliminary alert-response and safety framework for future clinical testing of the RAS. This framework will be developed with clinicians, people with lived experience, carers, emergency service representatives, and suicide prevention associations. It will address how alerts should be routed, which professionals should be responsible for responding, what minimum clinical assessment should follow an alert, how alert thresholds should be selected to balance false positives and false negatives, and how alert burden can be minimized. The co-design process will also examine whether risk scores or risk categories should be shared with patients, how such information should be communicated to avoid deterministic or stigmatizing interpretations, and what additional safeguards are needed for vulnerable groups, including people experiencing homelessness, poverty, limited access to health care records, or police custody.

Given that universal suicide risk screening can be considered a complex intervention—due to the number of components involved (eg, the use of RAS and another screening tool involved and staff training), the interaction with varied contexts (eg, might require coordination across different health settings), and the behavioral change required (change in the clinical workflow and decision-making)—the implementation research will be guided by the Medical Research Council (MRC) framework for complex interventions [37]. The implementation research will begin by collaboratively exploring and defining core elements of implementation design, including the context, program theory, key stakeholders, uncertainties, and economic considerations, as recommended in MRC guidance. During this project, implementation research will focus on the co-design of the tool and on exploring potential implementation pathways, following MRC-based guidance for developing new complex interventions [54].

Core elements and relevant previous interventions will be initially proposed by the research team, and subsequently discussed and refined with the UAG (Figure 2) through a focus group. An initial assessment of usability will be conducted to inform future phases. This co-design process will lay the foundation for subsequent stages of implementation research, including feasibility testing, evaluation, and implementation planning.

**Figure 2:**
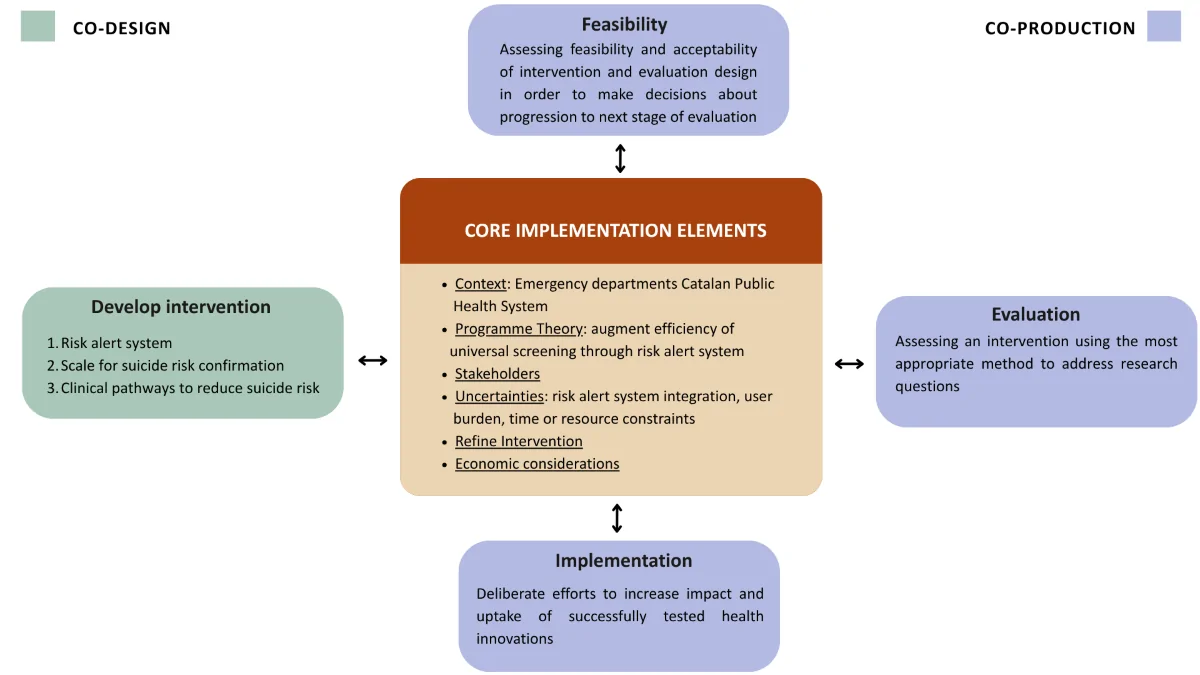
Medical Research Council framework for developing and evaluating complex interventions, adapted to the Clinical Risk Alert System for Suicide Risk Screening in Emergency Settings (CARES) project. Within the scope of the CARES project, only the co-design of the core implementation elements and the intervention itself will be carried out. RAS: risk alert system.

### Ethical Considerations

All research will be in line with the principles established by the Declaration of Helsinki and the Code of Ethics. The study protocol was approved by the Hospital del Mar Clinical Research Ethics Committee (2022/10325/I).

The processing of electronic registry data (ie, data harmonization followed by the development of prediction models) does not involve research with human subjects or the joint processing of personal data. The requirement of explicit informed consent for the use of this data has been waived given that such requirement would make any registry-based research impossible, and given that strict data protection regulations are in place to minimize risk for subject identification (eg, anonymization and/or registry data access through highly secure virtual environments).

During implementation research, when inviting eligible study participants, interviewers will provide a clear and thorough explanation of the informed consent process, ensuring that participants fully understand the study’s purpose, the voluntary nature of participation, potential risks, data confidentiality measures, and their right to withdraw at any time without consequence. Thus, the project will adhere to ethical standards for research involving vulnerable populations when recruiting participants with lived experience, also ensuring stipends for participation.

Because the current project develops and co-designs a RAS prototype but does not deploy the system for real-time clinical use, no automated suicide risk alerts will be generated in routine ED care during this study. Accordingly, this protocol does not involve clinical decisions, interventions, or restrictions of individual rights triggered by RAS-generated alerts. Instead, the co-design process will be used to define the ethical and safety requirements that must be met before future feasibility, implementation, or effectiveness studies. These requirements will include who should receive and respond to alerts, the clinical pathway following an alert, procedures for managing false-positive and false-negative alerts, whether and how risk information should be communicated to patients, and safeguards for vulnerable groups.

## Results

### Current Study Status

The CARES project was funded in March 2023. The project uses linked electronic registry data from Catalonia covering 2014-2019, including 2,982,736 eligible ED index visits from 603,098 patients. The first UAG meeting was held in December 2024. As of May 2026, the project is developing the initial prediction models and software backend, while iteratively refining the frontend through UAG meetings. Data analysis is ongoing, and the main results are expected to be published in spring 2027.

### Findings From the First UAG

Effective integration of a RAS for universal suicide risk screening in EDs requires careful alignment of its design and implementation with the operational realities and priorities of end users. To this end, an initial UAG meeting was held in December 2024. The discussion centered on 2 main domains: the technical and functional design of the RAS, and the definition of clinical pathways to enable its systematic use within routine care.

Regarding RAS design, participants highlighted substantial limitations associated with the use of EHR data for developing prediction models and generating risk alerts. These included the absence of key predictor variables such as social determinants and gender identity, as well as underreporting of psychiatric diagnoses and self-harm. Participants also noted the limited availability of data for vulnerable populations, such as individuals experiencing poverty, homelessness, or lacking digital or medical records, which raises concerns about their potential exclusion from risk detection efforts.

Stakeholders emphasized the importance of a risk stratification and visualization approach that supports clinical decision-making in a practical and actionable way, without rendering the assessment overly deterministic. It was noted that the RAS should be clearly framed as a clinical support tool rather than a diagnostic instrument or suicide risk scale. Stakeholders showed interest in a short-term prediction horizon (eg 1 week) to optimize utility in emergency settings, with separate modeling of NLISH and suicide. In general EDs, sensitivity should be prioritized to minimize false negatives, whereas in psychiatric emergency settings, specificity should be increased to mitigate alert fatigue. The use of an external dataset (Hospital del Mar Information System) was proposed as a validation strategy.

The need to define clear alert thresholds and align them with existing suicide prevention protocols, such as the CSRC surveillance program, was also highlighted. This program requires that all Catalan residents presenting with suspected suicide risk in public health care settings receive a face-to-face psychiatric evaluation, with high-risk individuals offered a follow-up mental health visit within 10 days and a phone call after 30 days to facilitate care access. There was debate about whether to share risk results with patients; some stakeholders supported this as a means to enhance engagement and empowerment, while others warned that such information might be perceived as definitive or unchangeable. The importance of transparency in model development and performance was consistently emphasized.

Simultaneously, stakeholders engaged in robust discussion regarding the optimal integration of a suicide RAS within existing clinical workflows in EDs. The absence of a clearly defined alert response protocol was identified as a risk factor for clinician burden and diminished alert credibility. Concerns were expressed about the potential for persistent alerts in patients with frequent emergency visits, highlighting the need for a system that dynamically reflects changes in clinical status. Significant feasibility concerns were raised about the challenges of real-time implementation of the RAS. These concerns are particularly relevant given that the initial RAS prototype would rely on manual entry of predictor variables, requiring data collection during triage. This introduces practical limitations related to time constraints and the availability of clinical personnel. Deliberations focused on the appropriate timing and setting for data collection (if the RAS cannot be integrated into the information system), including whether alerts should be generated during triage, subsequent clinical encounters, or a dedicated assessment phase, as well as the design of downstream procedures following risk detection. Pilot implementation will therefore require additional staffing or workload management. Alternative settings such as primary care and telephone emergency services were proposed as more viable environments for data collection. Stakeholders also underscored the importance of training and sensitization to promote effective uptake and use of the tool among clinical staff.

Additionally, stakeholders emphasized the importance of adapting the system to address the specific needs of vulnerable populations, including individuals experiencing extreme poverty or those in police custody, whose clinical trajectories may differ markedly from the general ED population. The need for gender-sensitive approaches in both risk assessment and postalert interventions was also highlighted. It was recommended that the presence of patient companions be systematically recorded, given their potential role in postalert care planning. Managing alerts for detained individuals was identified as particularly complex, requiring dedicated ethical and procedural protocols. Finally, stakeholders called for greater clarity regarding informed consent, data sharing practices, and mechanisms for dynamically updating risk status.

## Discussion

The integration of a machine learning–driven RAS within EDs presents promising opportunities to enhance the efficiency of suicide risk screening beyond traditional clinical assessments. Given suicide’s substantial public health burden and the fact that many at-risk individuals present to EDs without self-harm–related chief complaints, CARES aims to lower the practical threshold for systematic detection by enabling focused, data-informed targeted assessments.

Previous work has shown that integrating universal suicide risk screening protocols in EDs could improve detection and enable timely intervention. In the United States, several national health agencies have supported this approach. The Joint Commission, through its National Patient Safety Goal (NPSG.15.01.01), requires suicide risk screening in hospitals for patients treated for behavioral health conditions, including those in EDs. However, targeted screening among only those with known behavioral health disorders will miss many adults and children at risk [7]. Several large-scale studies have demonstrated the feasibility and effectiveness of universal suicide risk screening in ED [22-25]. The Emergency Department Safety Assessment and Follow-Up Evaluation study demonstrated that implementing universal screening protocols in ED settings across 8 hospitals was scalable and sustainable, substantially increased suicide risk screening assessments conducted by staff compared to treatment as usual (from 26% to 84%), and doubled the probability of suicide risk detection, from 2.9% to 5.7% [23]. Similarly, the Parkland Health & Hospital System program showed that universal suicide risk screening can be effectively implemented at scale, increasing positive screening rates from 1.6% to 6.3% while successfully managing the associated clinical burden [24]. These findings support the scalability of universal screening in busy ED environments. See Multimedia Appendix 1 for a detailed literature review of universal suicide risk screening in EDs, including evidence on feasibility and key implementation barriers, such as resource constraints and the capacity to respond to increased detection rates [55-57]. The appendix also provides an overview of brief, validated suicide risk screening instruments that could be used for targeted assessment, including the C-SSRS [58] and the ASQ [21].

The CARES project highlights several critical challenges in leveraging routinely collected electronic health data, including underreporting of psychiatric conditions and self-harm events, and the absence of key social determinants that may influence risk. At the same time, the availability of large-scale, population-based linked registry data in Catalonia provides a strong foundation for developing and validating clinically useful prediction models, and offers a unique opportunity to build a scalable tool aligned with real-world care pathways. This underscores the need for a subsequent risk assessment with a more holistic approach that incorporates social variables—not available in current EHR systems in Catalonia—given their potential impact on suicide risk. In CARES, this will be addressed by positioning the RAS as a first-step alert that prompts brief validated screening (eg, ASQ or C-SSRS) and clinician-led assessment, rather than as a stand-alone diagnostic instrument. Brief validated suicide risk screening instruments exist [59]. Multimedia Appendix 1 provides an overview of brief, validated suicide risk screening instruments that could be used for targeted assessment, including the C-SSRS [58] and the ASQ [21].

Stakeholders also underscored the need for a risk visualization approach that is informative yet nondeterministic, clear clinical protocols following alerts, and context-sensitive implementation strategies that consider population diversity. Accordingly, CARES will prioritize interpretable model outputs and user-centered visualizations (eg, transparent risk stratification and key contributing factors) to support clinical judgment and actionable decision-making. Prioritizing specificity in psychiatric emergency settings was suggested to reduce alert fatigue. Consistent with this, model selection and thresholding in CARES will not rely solely on area under the curve, but will explicitly consider clinically meaningful trade-offs (eg, sensitivity, specificity, PPV, workload implications, and alert fatigue) tailored to different ED contexts.

The participatory approach with end users revealed essential practical considerations for embedding RAS into clinical workflows, including defining clear protocols for alert response and optimizing the timing of risk assessments within emergency care encounters. The potential inability to integrate RAS into hospital electronic systems was identified as a major barrier to real-time implementation, as it would require manual entry of predictor variables during high-pressure moments such as triage. To mitigate this, CARES will evaluate implementation options that minimize manual data entry (eg, prioritizing parsimonious predictor sets and staged assessment workflows) and will use the co-design process to identify feasible points in the ED pathway for data capture and response. Stakeholders also highlighted the importance of training, ethical guidelines for data use, and strategies to mitigate the burden on clinicians. These elements could be incorporated into a structured implementation package co-designed with stakeholders (eg, training materials, response protocols, and guidance on communicating risk).

These insights affirm that successful adoption of suicide risk prediction tools depends not only on technical performance but also on addressing operational, ethical, and contextual factors through collaborative design and iterative refinement. The CARES project lays important groundwork for the use of machine learning–based tools in the implementation and improvement of universal suicide risk screening protocols within the ED. By combining large-scale registry data, clinically interpretable modeling, user-centered risk communication, and co-designed implementation strategies, CARES aims to deliver a feasible prototype that can be progressively integrated into routine emergency care. Ultimately, the project seeks to support timely, targeted assessment and intervention among ED patients who might otherwise remain undetected, thereby strengthening the foundations for scalable suicide prevention in Catalonia.

## Data Availability

The primary data, including health care, mortality, and administrative records, were provided by a third party, the Agency for Quality and Assessment of Catalonia (*Agència de Qualitat i Avaluació Sanitàries de Catalunya* [AQuAS]), under the Data Analytics Program for Health Research and Innovation (*Programa d’Analítica de Dades per a la Recerca i la Innovació en Salut* [PADRIS]) framework. Access to these data is restricted and must comply with PADRIS’s legal and ethical requirements. Interested parties can obtain access to the data, code, and documentation upon request, in accordance with the agreement’s provisions. The minimum dataset needed to replicate the analyses underlying this study, including the anonymized individual-level registry data, data dictionaries, and statistical code, is available upon reasonable request from the corresponding author, provided (1) the purpose is to replicate our analysis and results without additional investigator support, (2) access is granted following approval of a brief proposal and the signing of a data access agreement, and (3) the request aligns with the terms of our agreement with PADRIS/AQuAS and is approved by the PADRIS legal representative.

## Appendix

Multimedia Appendix 1 Literature review.

Multimedia Appendix 2 Sampling and weighting procedure.

Multimedia Appendix 3 Fundació la Marató de TV3 and the Secretaria d’Universitats i Recerca del Departament d’Economia i Coneixement of the Generalitat de Catalunya AGAUR 2021 (SGR 00624); and CIBER of Epidemiology & Public Health (CIBERESP; CB06/02/0046)

## Abbreviations

ASQ: Ask Suicide-Screening Questions
CARES: Clinical Risk Alert System for Suicide Risk Screening in Emergency Settings
CSRC: Catalonia Suicide Risk Code
C-SSRS: Columbia–Suicide Severity Rating Scale
ED: emergency department
EHR: electronic health record
GUI: graphical user interface
ICD: International Classification of Diseases
ICD-10: International Classification of Diseases, Tenth Revision
ICD-10-CM: International Classification of Diseases, Tenth Revision, Clinical Modification
ICD-9-CM: International Classification of Diseases, Ninth Revision, Clinical Modification
INE: National Statistics Institute (Instituto Nacional de Estadística)
MRC: Medical Research Council
NLISH: nonlethal intentional self-harm
PPV: positive predictive value
RAS: risk alert system
UAG: user advisory group
WHO: World Health Organization
