# Supplementary material for: Risk Alert System to Facilitate Universal Suicide Risk Screening in Emergency Care: Protocol for a Prediction Model and Software Development Study Using a Co-Design Approach
Source: JMIR Res Protoc. 2026 Sep 16;15:e100498. doi: 10.2196/100498 (PMC13628038; doi:10.2196/100498)
Supplement: Supplementary file 1 [file resprot_v15i1e100498_app1.pdf]

Literature Review

Emergency Departments as key medical settings for suicide risk detection

Emergency departments (EDs) are increasingly recognized as key settings for suicide risk detection within the general population [1–4], for several interrelated reasons. First, empirical evidence suggests that a significant proportion of individuals who die by suicide had presented to an ED in the months or the year preceding their death [5], yet were not screened for suicide risk during these encounters. Data from the United States and the United Kingdom indicate that between 38.8% and 59.9% of individuals who died by suicide had visited an ED within the year prior to their death [6,7]. Notably, while 38.8% received a diagnosis of self-harm, often during their final ED visit [6,7], between 42.2% and 60.3% were not diagnosed with a mental health or substance use disorder at any ED visit during the preceding year [8,9]. This pattern suggests that many at-risk individuals may not have undergone comprehensive mental health or suicide risk evaluations. ED presentations prior to suicide were more prevalent among females, individuals aged 40 or older, and those who died by non-violent means, with no consistent associations found for socioeconomic variables such as income or education [9]. Additionally, 28.2% had made three or more ED visits, and 21.0% had presented to multiple EDs within the year before their death [6]. Predictors of suicide shortly after a final ED visit included alcohol and drug misuse, as well as unemployment [6].

Second, suicidal thoughts are often not recognized without the implementation of standardized assessments. Estimates suggest that between 4.6% and 11.6% of adult patients presenting to the ED for non-psychiatric reasons report suicidal ideation when actively screened [10–12]; at paediatric emergency departments this is 3.6-5.7% [13–16]. Patients identified through active screening were frequently found to exhibit symptoms of mood, anxiety, or substance use disorders, particularly depression [10] and were more likely to be single, unemployed, low-income, or have limited educational attainment [11]. Detected patients also included patients explicitly reporting planning suicide, or having recent suicide attempts. Patients with suicidal ideation at the ED not only have a 10-fold higher risk for death by suicide, but also substantially increased risk of accidental and alcohol- and drug-related death [4]. Interestingly, evidence from paediatric ED studies suggest that over half (53%) of patients with psychiatric presenting complaints who screened positive did not present to the ED with suicide-related complaints [1], suggesting that systematic assessments among these patients is also warranted.

Third, ED frequently serve as the primary, and often sole, point of contact with the healthcare system for individuals experiencing a crisis [17,18]. A considerable proportion of individuals who die by suicide live in socioeconomically disadvantaged areas [19], underscoring the role of EDs in reaching populations with limited access to outpatient mental health services. The unique position of EDs to engage vulnerable groups has been well documented due to their improved accessibility for high-risk groups, including men and socioeconomically vulnerable populations [20–23]. A study involving universal suicide risk screening found that a higher proportion of individuals who screened positive were young, male, non-Hispanic White, had a mental health diagnosis, were single or divorced, and reported housing insecurity [24]. Importantly, these individuals were less likely to seek primary care and more likely to present to the ED, consistent with literature describing EDs as entry points for patients with suicide risk [24]. Moreover, EDs display smaller gender disparities in healthcare utilization compared to other medical settings [21], further emphasizing their strategic relevance in suicide risk detection and intervention.

Universal Suicide Risk Screening at the Emergency Department

The evidence discussed in the previous section suggests that integrating universal suicide risk screening protocols in EDs could improve detection and enable timely intervention. In the United States, several national health agencies have supported this approach. The Joint Commission, through its National Patient Safety Goal (NPSG.15.01.01), requires suicide risk screening in hospitals for patients treated for behavioural health conditions, including those in EDs. However, targeted screening among only those with known behavioural health disorders will miss many adults and children at risk [2]. A retrospective cohort study of patients aged 18 or less visiting the ED showed that, adjusting for demographic characteristics and baseline presenting problem, positive suicide risk screens are significantly associated with suicide-related outcomes (i.e., ideation, attempt, or suicide) both among detected patients using a targeted protocol (screening restricted to those with behavioral and psychiatric presenting problems) or using universal screening (hazard ratio, 4.8 and 6.8, respectively) [13]. The Substance Abuse and Mental Health Services Administration (SAMHSA) recommends integrating suicide risk screening into routine ED care as a core component of comprehensive prevention strategies. Similarly, the Veterans Health Administration (VHA) mandates systematic screening through its Risk ID programme, which requires all veterans presenting to ED or outpatient services to be assessed for suicide risk.

Several large-scale studies have demonstrated the feasibility and effectiveness of universal suicide risk screening in ED [24–27]. The ED-SAFE study demonstrated that implementing universal screening protocols in ED settings across 8 hospitals was scalable and sustainable, substantially increased suicide risk screening assessments conducted by staff compared to treatment as usual (from 26% to 84%) and doubling the probability of suicide risk detection, from 2.9% to 5.7% [25]. Similarly, the Parkland Health & Hospital System (PHHS) programme showed that universal suicide risk screening can be effectively implemented at scale, increasing positive screening rates from 1.6% to 6.3% while successfully managing the associated clinical burden [26]. Paediatric studies have shown that brief screening conducted while patients wait for a physician did not prolong ED stay, suggesting that such approaches can be integrated without disrupting workflows [15]. These findings support the scalability of universal screening in busy ED environments.

Despite promising findings, several limitations and implementation barriers remain in the current evidence on universal suicide risk screening in emergency departments. Recent large-scale studies in adult populations, while valuable, did not differentiate patients by admission reason (psychiatric vs. non-psychiatric), limiting the applicability of results to specific clinical contexts. Moreover, no standardized procedure has yet been established that ensures efficient screening without disrupting ED workflows [28]. This gap is closely aligned with concerns voiced by clinicians regarding time and resource constraints, the lack of adequate screening tools, and the ability to respond to increased detection rates [29,30]. Many studies also relied on research staff to administer screenings, which may have led to higher participation rates than would be observed in routine clinical practice [28]. Differences in screening outcomes also highlight the challenge of consistent risk classification. For example, a study using the Columbia–Suicide Severity Rating Scale (C-SSRS) in over 500,000 ED visits found that 2.2% of all visits screened positive for suicide risk, and 47.4% of those positive screens were classified as high risk [24]. Another study across more than one million ED encounters in the U.S. healthcare system found that 1.83–3.93% of visits per month

screened positive overall, of which only 10.7% were classified as high risk [27]. Nonetheless, strategic planning, such as augmenting social work staffing and adopting tiered clinical responses, has shown promise in addressing these challenges [26].

Importantly, although universal suicide risk screening faces challenges such as stigma, unfamiliarity between patients and clinicians, and time constraints [31], it is generally acceptable to both patients and clinicians, especially when implemented with adequate planning. Among paediatric patients presenting with non-psychiatric complaints at the ED, 96% support suicide screening, with both patients and parents being willing to participate and discuss past suicidal thoughts and behaviours, with many adolescents expressing relief at being asked such questions. While some parents initially hesitated, especially with younger children, most consented when the importance of screening was explained [15]. While there may be resistance initially among clinicians to implement universal screening, this diminished over time, with staff reporting increased confidence in patient safety after programme rollout. Involving ED staff in developing the screening protocols, using a quality improvement model, as in the ED-SAFE study [25], fostered engagement and acceptance. Performance incentives and feedback loops were also critical in increasing compliance and perceived value among staff. Notably, self-administered computerized screenings have demonstrated to further increase acceptability from both patients and clinicians, facilitating higher disclosure on sensitive topics and improving efficiency through more detailed post-test assessments [14,31].

In light of these workflow constraints and implementation challenges, technology-assisted approaches may function as potential complements to universal screening strategies. Attitudes toward automated suicide risk prediction models are generally positive, with practitioners expressing growing trust in their potential to support clinical decision-making and treatment planning [32]. Nonetheless, their use raises specific challenges, including concerns about liability, dependence on the quality and completeness of electronic health records (EHR) data, and the risk of alert fatigue or desensitization over time. Practitioners highlighted the value of features such as the identification of patient-specific risk factors and the ability to monitor changes in risk scores over time. Systematic training was widely regarded as crucial to ensure appropriate and effective use in practice [32]. While RAS may offer a promising solution to address time constraints and enhance the quality of assessments, it is equally important to explore effective implementation strategies. In this context, co-design can serve as a valuable methodology to tailor the screening process to real-world conditions.

Suicide screening assessment tools

The effectiveness of universal suicide risk screening in emergency departments could be enhanced through the integration of machine learning–based predictive models that automatically analyse patients' EHR [33]. These models can identify individuals at elevated risk and flag them for further evaluation using validated suicide risk screening instruments. This approach enables healthcare providers to concentrate valuable clinical resources on patients most in need of suicide risk assessments.

Brief validated suicide risk screening instruments exist. Some of the current screening tools consist of a few questions that can help decide if a more extensive evaluation is required. Thom et al. (2020) [34] evaluated several brief scales with fewer than 10 questions each, including the Suicidal Behaviours Questionnaire (SBQ), the C-SSRS, the Ask Suicide-Screening Questions (ASQ), the Suicide Affect-Behaviour-Cognition Scale, the Patient Safety Screener-3, and the Patient Health Questionnaire item 9 (PHQ-

9). Among these, the C-SSRS and ASQ are the ones recommended by the authors, while highlighting their limitations.

The ASQ questionnaire was developed to assess suicide risk in children and young adults and has been validated in EDs for patients presenting with either medical/surgical or psychiatric chief concerns. This four-question screening tool demonstrates a sensitivity of 96.9% and a specificity of 87.6% [16]. It can be administered in under two minutes without requiring specialized training, making it well-suited for universal screening initiatives [13,35]. Alongside the C-SSRS, the ASQ is recommended as an initial step to determine whether a comprehensive suicide risk assessment is needed [36]. The C-SSRS is unique for its application across both paediatric and adult populations and is known for its high sensitivity and specificity (94-100% and 99-99.4% respectively) [34]. However, it has limitations: it is not validated among medical patients, is too lengthy for brief assessments, and may not fully capture the range of possible combinations of suicidal ideation, method, intent, and plan that present in clinical practice [37]. Moreover, a 2022 systematic review and meta-analysis evaluated suicide screening tools using the likelihood ratio, which measures how much more (or less) likely it is that a patient that died by suicide would have that test result compared to a patient that did not die by suicide [38,39]. The review found that most instruments are not particularly effective at detecting suicide risk, with the C-SSRS being one of the few tools that reliably identifies potential suicide cases specifically in emergency settings [39]. A growing body of research has demonstrated that machine learning-based prediction models can be developed to stratify patients according to risk for self-harm and suicide. The growing interest in machine learning and artificial intelligence for suicide prediction [40] is driven by their ability to analyse complex interactions among multiple risk factors. Machine learning methods have already demonstrated superior predictive capacity compared to traditional theory-driven approaches [41]. Studies evaluating machine learning models often report area under the receiver operating characteristic curve (AUC) values in the range of 0.80–0.90, whereas logistic regression models in comparable settings more frequently demonstrate AUCs below 0.70 [42,43].

Recent research has demonstrated that machine learning approaches can substantially enhance suicide prediction, outperforming traditional clinician assessments and screening tools alone [44,45]. Nock et al. [33] conducted a prospective study of 1,818 psychiatric emergency department patients and found that clinician judgment alone had limited predictive value for suicide attempts (AUC = 0.60–0.67), whereas models incorporating patient self-reports and EHRs achieved significantly better performance (AUC up to 0.79) using ensemble ML techniques, even with brief self-report instruments and minimal EHR inputs. Similarly, Wilimitis et al. [46] evaluated over 120,000 clinical encounters and showed that combining the Columbia Suicide Severity Rating Scale with a real-time EHR-based ML model (VSAIL) yielded superior predictive validity (AUC = 0.87 for suicide attempts) compared to either method alone, reinforcing the value of hybrid approaches for clinical deployment. Expanding beyond ED settings, Sheu et al. [47] developed a landmark modelling framework using EHR data across outpatient, psychiatric ED, and inpatient populations. Their ML-based survival models achieved robust performance (AUC = 0.74–0.93) across diverse settings and prediction windows, while addressing key limitations such as temporal bias and healthcare utilization confounding. Complementing these findings in a veteran population, Kennedy et al. [48] demonstrated that suicide risk in over 800,000 US Army soldiers could be reliably predicted for up to 10 years after service separation using

administrative data and lasso-based models, with the highest-risk decile accounting for nearly half of all observed suicides (AUC = 0.72–0.87). Collectively, these studies support the integration of ML models into clinical workflows to improve suicide risk stratification and enable more targeted, data-driven preventive interventions.

Co-creation in suicide interventions

The involvement of stakeholders in designing clinical interventions has gained increasing recognition as a means of bridging the knowledge–action gap. Co-creation—an umbrella concept encompassing co-design (collaborative planning) and co-production (stakeholder involvement in implementation)—has emerged as a participatory approach that enhances innovation, facilitates implementation, increases initiative success, and strengthens equity in health initiatives [49–51]. In suicide prevention, this approach is particularly relevant given the complexity and sensitivity of care contexts. Many individuals presenting with emotional distress, especially in emergency settings, report negative care experiences, underscoring the need for services that meaningfully incorporate the perspectives of both patients and providers. Co-created interventions in suicide prevention have shown promise in addressing these challenges, and recent reviews highlight growing applications of co-design in self-harm interventions, including those implemented in emergency departments [52].

Owens et al. developed an SMS intervention to reduce self-harm repetition, where service users actively influenced the design, advocating for personalised messages [53]. Co-creation fundamentally reshaped the design of this text-based intervention by centring the voices of people with lived experience of self-harm. Initial plans for generic or segmented messages were rejected by service users, who emphasized the potential harm of receiving impersonal or mismatched content. Through a series of participatory workshops, users led the development of a system that allows individuals to author and retrieve their own self-efficacy messages during crises. This shift—from a broadcast model to a personalised, user-initiated one—not only made the intervention more acceptable and empowering but also aligned it with recovery-oriented principles of autonomy and self-management.

MacLean et al. proposed the BEACON trial, which evaluated a combined therapy intervention (in-person therapy plus a smartphone app) for men presenting to the emergency department (ED) after self-harm [54]. The study involved patient participation in two ways: as co-investigators and by aiming to create a community of individuals with lived experience in suicide prevention. Co-creation led to the integration of face-to-face therapy with a purpose-built smartphone app tailored specifically for men, enhancing both accessibility and engagement. Additionally, challenges in maintaining formal advisory structures informed a shift toward more flexible, inclusive practices such as allowing space for “testament”—personal narratives that centre service users’ voices—and acknowledging the dynamic nature of mental wellness by permitting partners to withdraw or re-join as needed. These collaborative processes not only strengthened the intervention itself but also shaped a more ethical and empowering research environment.

Similarly, Shin et al. used collaborative design to develop the *Hope* app, a free, evidence-based mobile tool designed to deliver suicide safety planning interventions digitally within psychiatric emergency departments. It provides users with accessible, customizable features such as coping strategies, emergency contacts, and options to share safety plans with their support network. Co-creation with clinicians, patients, and families enabled the development of implementation strategies that were contextually

relevant and practically feasible. Through structured co-design sessions, stakeholders prioritized barriers to address and selected behaviour change techniques aligned with real-world clinical workflows. The process fostered shared ownership, enhanced buy-in, and incorporated digital equity considerations, such as supporting users with low digital literacy. As a result, the intervention's integration into routine care was more acceptable, sustainable, and responsive to the needs of all user groups [55].

Shah et al. employed a collaborative approach to involve both patients and professionals in the development of the Assured intervention, a brief, person-centred psychological approach combining narrative-based assessment and collaborative safety planning with rapid, solution-focused follow-up. It fosters therapeutic alliance and empowers patients to manage future crises through personalised strategies and consistent practitioner support [56]. Co-creation significantly enhanced the Assured intervention by centring the experiences and needs of people who self-harm in its design and delivery. Patient feedback directly informed key components such as narrative-based assessments and personalised safety planning, replacing standard checklist approaches with compassionate, empowering conversations. This collaborative process improved therapeutic engagement, fostered trust between patients and practitioners, and led to a more humanized, responsive model of care. Practitioners also adapted their methods based on real-time feedback, making the intervention more flexible and patient-led. As a result, co-creation helped shift crisis care from reactive risk management to proactive, person-centred support.

The benefits of co-creation (including co-design and co-production) are clear: it fosters collaboration, improves interventions' relevance, and enhances the care experience. For this reason, it is integral to the success of this project. Implementing a RAS for universal suicide risk screening in EDs may face challenges, mostly related to resource constraints. By applying co-creation methodologies, we can address these barriers and ensure the system's integration into clinical practice. This approach can significantly contribute to the state-of-the-art, as no existing project has explored suicide risk detection via automated screening in EDs through co-creation methodology.

References

1. Ballard ED, Cwik M, Van Eck K, Goldstein M, Alfes C, Wilson ME, Virden JM, Horowitz LM, Wilcox HC. Identification of At-Risk Youth by Suicide Screening in a Pediatric Emergency Department. Prev Sci 2017 Feb 1;18(2):174–182. PMID:27678381

2. Goldstein Grumet J, Boudreaux ED. Universal Suicide Screening Is Feasible and Necessary to Reduce Suicide. Psychiatr Serv American Psychiatric Publishing; 2023 Jan;74(1):81–83. doi: 10.1176/appi.ps.202100625

3. Melhem N, Moutier CY, Brent DA. Implementing Evidence-Based Suicide Prevention Strategies for Greatest Impact. Focus Am Psychiatr Publ 2023 Apr;21(2):117–128. PMID:37201145

4. Ross E, Murphy S, O'Hagan D, Maguire A, O'Reilly D. Emergency department presentations with suicide and self-harm ideation: a missed opportunity for intervention? Epidemiol Psychiatr Sci 2023 Apr 18;32:e24. PMID:37069827

5. Vasiliadis H-M, Ngamini-Ngui A, Lesage A. Factors associated with suicide in the month following contact with different types of health services in Quebec. Psychiatr Serv Wash DC 2015 Feb 1;66(2):121–126. PMID:25270296

6. Da Cruz D, Pearson A, Saini P, Miles C, While D, Swinson N, Williams A, Shaw J, Appleby L, Kapur N. Emergency department contact prior to suicide in mental health patients. Emerg Med J EMJ 2011 June;28(6):467–471. PMID:20660941

7. Gairin I, House A, Owens D. Attendance at the accident and emergency department in the year before suicide: retrospective study. Br J Psychiatry J Ment Sci 2003 July;183:28–33. PMID:12835240

8. Ahmedani BK, Westphal J, Autio K, Elsiss F, Peterson EL, Beck A, Waitzfelder BE, Rossom RC, Owen-Smith AA, Lynch F, Lu CY, Frank C, Prabhakar D, Braciszewski JM, Miller-Matero LR, Yeh H-H, Hu Y, Doshi R, Waring SC, Simon GE. Variation in patterns of health care before suicide: A population case-control study. Prev Med 2019 Oct 1;127:105796. doi: 10.1016/j.ypmed.2019.105796

9. Ahmedani BK, Simon GE, Stewart C, Beck A, Waitzfelder BE, Rossom R, Lynch F, Owen-Smith A, Hunkeler EM, Whiteside U, Operskalski BH, Coffey MJ, Solberg LI. Health care contacts in the year before suicide death. J Gen Intern Med 2014 June;29(6):870–877. PMID:24567199

10. Claassen CA, Larkin GL. Occult suicidality in an emergency department population. Br J Psychiatry J Ment Sci 2005 Apr;186:352–353. PMID:15802695

11. Ilgen MA, Walton MA, Cunningham RM, Barry KL, Chermack ST, De Chavez P, Blow FC. Recent suicidal ideation among patients in an inner city emergency department. Suicide Life Threat Behav 2009 Oct;39(5):508–517. PMID:19929151

12. Roaten K, Horowitz LM, Bridge JA, Goans CRR, McKintosh C, Genzel R, Johnson C, North CS. Universal Pediatric Suicide Risk Screening in a Health Care System: 90,000 Patient Encounters. J Acad Consult-Liaison Psychiatry 2021;62(4):421–429. PMID:34219656

13. DeVylder JE, Ryan TC, Cwik M, Wilson ME, Jay S, Nestadt PS, Goldstein M, Wilcox HC. Assessment of Selective and Universal Screening for Suicide Risk in a Pediatric Emergency Department. JAMA Netw Open 2019 Oct 25;2(10):e1914070. doi: 10.1001/jamanetworkopen.2019.14070

14. Fein JA, Pailler ME, Barg FK, Wintersteen MB, Hayes K, Tien AY, Diamond GS. Feasibility and effects of a Web-based adolescent psychiatric assessment administered by clinical staff in the pediatric emergency department. Arch Pediatr Adolesc Med 2010 Dec;164(12):1112–1117. PMID:21135339

15. Horowitz L, Ballard E, Teach SJ, Bosk A, Rosenstein DL, Joshi P, Dalton ME, Pao M. Feasibility of Screening Patients With Nonpsychiatric Complaints for Suicide Risk in a Pediatric Emergency Department. Pediatr Emerg Care 2010 Nov;26(11):787–792. PMID:20944511

16. Horowitz LM, Bridge JA, Teach SJ, Ballard E, Klima J, Rosenstein DL, Wharff EA, Ginnis K, Cannon E, Joshi P, Pao M. Ask Suicide-Screening Questions (ASQ): A Brief Instrument for the Pediatric Emergency Department. Arch Pediatr Adolesc Med 2012 Dec 1;166(12):1170–1176. doi: 10.1001/archpediatrics.2012.1276

17. Asarnow JR, Babeva K, Horstmann E. The Emergency Department: Challenges & Opportunities for Suicide Prevention. Child Adolesc Psychiatr Clin N Am 2017 Oct;26(4):771–783. PMID:28916013

18. Larkin GL, Beautrais AL. Emergency departments are underutilized sites for suicide prevention. Crisis 2010;31(1):1–6. PMID:20197251

19. O'Neill S, Graham B, Ennis E. Emergency department and hospital care prior to suicide: A population based case control study. J Affect Disord 2019 Apr 15;249:366–370. doi: 10.1016/j.jad.2019.02.052

20. Flood C, Sheehan K, Crandall M. Predictors of Emergency Department Utilization Among Children in Vulnerable Families. Pediatr Emerg Care 2017 Dec;33(12):765–769. PMID:26959517

21. King CA, Horwitz A, Czyz E, Lindsay R. Suicide Risk Screening in Healthcare Settings: Identifying Males and Females at Risk. J Clin Psychol Med Settings 2017 Mar;24(1):8–20. PMID:28251427

22. Venkatesh AK, Mei H, Shuling L, D'Onofrio G, Rothenberg C, Lin Z, Krumholz HM. Cross-sectional Analysis of Emergency Department and Acute Care Utilization Among Medicare Beneficiaries. Acad Emerg Med Off J Soc Acad Emerg Med 2020 July;27(7):570–579. PMID:32302034

23. Wilson KM, Klein JD. Adolescents Who Use the Emergency Department as Their Usual Source of Care. Arch Pediatr Adolesc Med 2000 Apr 1;154(4):361–365. doi: 10.1001/archpedi.154.4.361

24. Dillon EC, Huang Q, Deng S, Li M, de Vera E, Pesa J, Nguyen T, Kiger A, Becker DF, Azar K. Implementing universal suicide screening in a large healthcare system's hospitals: rates of screening, suicide risk, and documentation of subsequent psychiatric care. Transl Behav Med 2023 Apr 1;13(4):193–205. doi: 10.1093/tbm/ibac117

25. Boudreaux ED, Camargo CA, Arias SA, Sullivan AF, Allen MH, Goldstein AB, Manton AP, Espinola JA, Miller IW. Improving Suicide Risk Screening and Detection in the Emergency Department. Am J Prev Med 2016 Apr;50(4):445–453. PMID:26654691

26. Roaten K, Johnson C, Genzel R, Khan F, North CS. Development and Implementation of a Universal Suicide Risk Screening Program in a Safety-Net Hospital System. Jt Comm J Qual Patient Saf 2018 Jan;44(1):4–11. PMID:29290245

27. Fertel BS, Pozuelo L, Kirschling S, Worley S, Simon EL, Muir M, Smalley CM. Universal suicide screening in emergency departments across a large healthcare system. Am J Emerg Med 2023 Oct;72:127–131. PMID:37523993

28. Cervantes PE, Seag DEM, Baroni A, Gerson R, Knapp K, Tay ET, Wiener E, Horwitz SM. Universal Suicide Risk Screening for Youths in the Emergency Department: A Systematic Review. Psychiatr Serv Wash DC 2022 Jan 1;73(1):53–63. PMID:34106741

29. Cronholm PF, Barg FK, Pailler ME, Wintersteen MB, Diamond GS, Fein JA. Adolescent depression: views of health care providers in a pediatric emergency department. Pediatr Emerg Care 2010 Feb;26(2):111–117. PMID:20093998

30. Habis A, Tall L, Smith J, Guenther E. Pediatric Emergency Medicine Physicians' Current Practices and Beliefs Regarding Mental Health Screening. Pediatr Emerg Care 2007 June;23(6):387. doi: 10.1097/01.pec.0000278401.37697.79

31. Babeva K, Hughes JL, Asarnow J. Emergency Department Screening for Suicide and Mental Health Risk. Curr Psychiatry Rep 2016 Nov;18(11):100. PMID:27671917

32. Bentley KH, Zuromski KL, Fortgang RG, Madsen EM, Kessler D, Lee H, Nock MK, Reis BY, Castro VM, Smoller JW. Implementing Machine Learning Models for Suicide Risk Prediction in Clinical Practice: Focus Group Study With Hospital Providers. JMIR Form Res 2022 Mar 11;6(3):e30946. PMID:35275075

33. Nock MK, Millner AJ, Ross EL, Kennedy CJ, Al-Suwaidi M, Barak-Corren Y, Castro VM, Castro-Ramirez F, Lauricella T, Murman N, Petukhova M, Bird SA, Reis B, Smoller JW, Kessler RC. Prediction of Suicide Attempts Using Clinician Assessment, Patient Self-report, and Electronic Health Records. JAMA Netw Open 2022 Jan 4;5(1):e2144373. PMID:35084483

34. Thom R, Hogan C, Hazen E. Suicide Risk Screening in the Hospital Setting: A Review of Brief Validated Tools. Psychosomatics 2020;61(1):1–7. PMID:31629482

35. Brent DA, Horowitz LM, Grupp-Phelan J, Bridge JA, Gibbons R, Chernick LS, Rea M, Cwik MF, Shenoi RP, Fein JA, Mahabee-Gittens EM, Patel SJ, Mistry RD, Duffy S, Melzer-Lange MD, Rogers A, Cohen DM, Keller A, Hickey RW, Page K, Casper TC, King CA, Pediatric Emergency Care Applied Research Network (PECARN). Prediction of Suicide Attempts and Suicide-Related Events Among Adolescents Seen in Emergency Departments. JAMA Netw Open 2023 Feb 1;6(2):e2255986. PMID:36790810

36. Brahmbhatt K, Kurtz BP, Afzal KI, Giles LL, Kowal ED, Johnson KP, Lanzillo E, Pao M, Plioplys S, Horowitz LM. Suicide Risk Screening in Pediatric Hospitals: Clinical Pathways to Address a Global Health Crisis. Psychosomatics 2019 Jan 1;60(1):1–9. doi: 10.1016/j.psym.2018.09.003

37. Giddens JM, Sheehan KH, Sheehan DV. The Columbia-Suicide Severity Rating Scale (C-SSRS): Has the "Gold Standard" Become a Liability? Innov Clin Neurosci 2014 Sept;11(9–10):66–80. PMID:25520890

38. Deeks JJ, Altman DG. Diagnostic tests 4: likelihood ratios. BMJ 2004 July 17;329(7458):168–169. PMID:15258077

39. Riblet NB, Matsunaga S, Lee Y, Young-Xu Y, Shiner B, Schnurr PP, Levis M, Watts BV. Tools to Detect Risk of Death by Suicide: A Systematic Review and Meta-Analysis. J Clin Psychiatry 2022 Nov 16;84(1):21r14385. PMID:36383739

40. Lejeune A, Glaz AL, Perron P-A, Sebti J, Baca-Garcia E, Walter M, Lemey C, Berrouiguet S. Artificial intelligence and suicide prevention: A systematic review. Eur Psychiatry 2022 Jan;65(1):e19. doi: 10.1192/j.eurpsy.2022.8

41. Schafer KM, Kennedy G, Gallyer A, Resnik P. A direct comparison of theory-driven and machine learning prediction of suicide: A meta-analysis. PloS One 2021;16(4):e0249833. PMID:33844698

42. Walsh CG, Ribeiro JD, Franklin JC. Predicting suicide attempts in adolescents with longitudinal clinical data and machine learning. J Child Psychol Psychiatry 2018;59(12):1261–1270. doi: 10.1111/jcpp.12916

43. Zheng L, Wang O, Hao S, Ye C, Liu M, Xia M, Sabo AN, Markovic L, Stearns F, Kanov L, Sylvester KG, Widen E, McElhinney DB, Zhang W, Liao J, Ling XB. Development of an early-warning system for high-risk patients for suicide attempt using deep learning and electronic health records. Transl Psychiatry Nature Publishing Group; 2020 Feb 20;10(1):1–10. doi: 10.1038/s41398-020-0684-2

44. Woodford R, Spittal MJ, Milner A, McGill K, Kapur N, Pirkis J, Mitchell A, Carter G. Accuracy of Clinician Predictions of Future Self-Harm: A Systematic Review and Meta-Analysis of Predictive Studies. Suicide Life Threat Behav 2019 Feb;49(1):23–40. PMID:28972271

45. Carter G, Milner A, McGill K, Pirkis J, Kapur N, Spittal MJ. Predicting suicidal behaviours using clinical instruments: systematic review and meta-analysis of positive predictive values for risk scales. Br J Psychiatry J Ment Sci 2017 June;210(6):387–395. PMID:28302700

46. Wilimitis D, Turer RW, Ripperger M, McCoy AB, Sperry SH, Fielstein EM, Kurz T, Walsh CG. Integration of Face-to-Face Screening With Real-time Machine Learning to Predict Risk of Suicide Among Adults. JAMA Netw Open 2022 May 2;5(5):e2212095. PMID:35560048

47. Sheu Y-H, Sun J, Lee H, Castro VM, Barak-Corren Y, Song E, Madsen EM, Gordon WJ, Kohane IS, Churchill SE, Reis BY, Cai T, Smoller JW. An efficient landmark model for prediction of suicide attempts in multiple clinical settings. Psychiatry Res 2023 May;323:115175. PMID:37003169

48. Kennedy CJ, Kearns JC, Geraci JC, Gildea SM, Hwang IH, King AJ, Liu H, Luedtke A, Marx BP, Papini S, Petukhova MV, Sampson NA, Smoller JW, Wolock CJ, Zainal NH, Stein MB, Ursano RJ, Wagner JR, Kessler RC. Predicting Suicides Among US Army Soldiers After Leaving Active Service. JAMA Psychiatry 2024 Dec 1;81(12):1215–1224. doi: 10.1001/jamapsychiatry.2024.2744

49. Pearce T, Maple M, McKay K, Shakeshaft A, Wayland S. Co-creation of new knowledge: Good fortune or good management? Res Involv Engagem 2022 Nov 29;8(1):65. PMID:36447281

50. Vargas C, Whelan J, Brimblecombe J, Allender S. Co-creation, co-design, co-production for public health – a perspective on definition and distinctions. Public Health Res Pract 2022;32(2). doi: 10.17061/phrp3222211

51. Fitzpatrick SJ, Lamb H, Stewart E, Gulliver A, Morse AR, Giugni M, Banfield M. Co-ideation and co-design in co-creation research: Reflections from the "Co-Creating Safe Spaces" project. Health Expect Int J Public Particip Health Care Health Policy 2023 Aug;26(4):1738–1745. PMID:37254844

52. Wright LC, Lopez Chemas N, Cooper C. Lived experience codesign of self-harm interventions: a scoping review. BMJ Open 2023 Dec;13(12):e079090. doi: 10.1136/bmjopen-2023-079090

53. Owens C, Farrand P, Darvill R, Emmens T, Hewis E, Aitken P. Involving service users in intervention design: a participatory approach to developing a text-messaging intervention to reduce repetition of self-harm. Health Expect Int J Public Particip Health Care Health Policy 2011 Sept;14(3):285–295. PMID:20860777

54. MacLean S, MacKie C, Hatcher S. Involving people with lived experience in research on suicide prevention. CMAJ Can Med Assoc J J Assoc Medicale Can 2018 Nov 7;190(Suppl):S13–S14. PMID:30404841

55. Shin HD, Zaheer J, Torous J, Strudwick G. Designing Implementation Strategies for a Digital Suicide Safety Planning Intervention in a Psychiatric Emergency Department: Protocol for a Multimethod Research Project. JMIR Res Protoc 2023 Nov 9;12:e50643. PMID:37943582

56. Shah N, O'Keeffe S, Hayward S, Suzuki M, McCabe R. Re-imagining crisis care: experiences of delivering and receiving the Assured brief psychological intervention for people presenting to Emergency Departments with self-harm. Front Psychiatry 2024;15:1271674. PMID:38600980
