# Supplementary material for: Risk Alert System to Facilitate Universal Suicide Risk Screening in Emergency Care: Protocol for a Prediction Model and Software Development Study Using a Co-Design Approach
Source: JMIR Res Protoc. 2026 Sep 16;15:e100498. doi: 10.2196/100498 (PMC13628038; doi:10.2196/100498)
Supplement: Supplementary file 2 [file resprot_v15i1e100498_app2.pdf]

Sampling and weighting procedure

The sampling design described here generates a population-based analytic dataset that is subsequently used in multiple studies with study-specific inclusion criteria. Sampling procedures were implemented in collaboration with the Data Analytics Program for Health Research and Innovation (PADRIS) [1]. All data extraction, linkage, and sampling operations were conducted centrally by PADRIS using pseudonymised individual-level identifiers.

Eligible population and data sources

The population eligible for inclusion in the sampling frame comprised all residents of Catalonia, Spain, who were alive at any point during the period from 1 January 2014 to 31 December 2019 (n = 8,662,155). The sampling frame was constructed using administrative population registries of individuals with access to publicly funded healthcare. In Catalonia, access to public healthcare is universal; therefore, these registries provide near-complete coverage of the resident population.

Sampling design overview

An overview of the sampling process, including case status, strata definition, numbers of eligible and selected individuals, selection probabilities, and inverse probability weights (IPWs), is provided in **Table 1**.

Using the population-based sampling frame, we employed an outcome- and exposure-enriched sampling design [2-6], analogous to a case-cohort study [7,8]. All eligible individuals were classified according to case status (case vs non-case; see section **Case identification** below) and a predefined hierarchical stratification variable capturing healthcare setting and mental disorder indicators (see section **Stratification and sampling of non-cases** below). All identified cases were included in the analytic dataset, while non-cases were sampled disproportionately across strata to enrich the analytic dataset for individuals characterised by mental disorder indicators across different healthcare settings. IPWs were subsequently assigned to enable population-representative analyses.

Case identification

Cases were defined as individuals with a recorded diagnosis or clinically confirmed episode of **suicidal ideation or self-harm**, identified using linked healthcare data from two complementary sources. Case status was determined by reviewing **all available information for each individual across the observation windows of the linked data sources**, noting that data availability differed by source.

First, routine administrative healthcare data were used to identify diagnoses of suicidal ideation or self-harm in four healthcare settings:

- **Primary care**, available for the period 2010–2019
- **Emergency care**, available for the period 2014–2019
- **General hospitalisations**, available for the period 2007–2019
- **Psychiatric hospitalisations**, available for the period 2008–2019

In routine electronic healthcare data, suicidal ideation and self-harm were identified using recorded ICD-9-CM and ICD-10(-CM) diagnostic and external cause codes (see **Table 2**).

Second, data from the **Catalonia Suicide Risk Code (CSRC)** registry were used to identify clinically confirmed episodes of suicidal ideation or intentional self-harm for the period **2014-2022**. The CSRC is an integrated, system-wide suicide prevention programme [9] within the Catalan public healthcare system that mandates a face-to-face psychiatric evaluation for individuals presenting with self-harm or suicide risk in any public healthcare setting.

An individual was classified as a case if **a diagnosis or episode of suicidal ideation or self-harm was recorded at any time within the observation window of any of these sources**, including:

- events occurring **before 2014** in data sources with earlier coverage (primary care and hospitalisation registries), and
- events occurring **after 2019** in the CSRC registry.

Using these criteria, a total of n = **93,599 individuals** in the eligible population were classified as cases (of those, n = 76,148 individuals had at least one episode of ideation or self-harm in the period 2014–2019). All identified cases were retained in the analytic dataset with a selection probability of 1 and assigned an IPW of 1. All other eligible individuals were classified as eligible non-cases.

Stratification and sampling of non-cases

To enable disproportionate sampling and enrichment of the analytic dataset for individuals with indicators of mental disorders and related patterns of healthcare use, the eligible population was stratified using a 15-level hierarchical stratification variable. This variable was defined by crossing healthcare setting with mental disorder indicator variables available in the central population register.

The 15 strata correspond exactly to the rows shown in **Table 1**. Stratification reflects whether individuals ever met the corresponding healthcare setting and mental disorder indicator criteria during the period 2014–2019. Strata were made mutually exclusive through hierarchical assignment: each individual was assigned to the highest applicable stratum according to a predefined hierarchical ordering of strata, as detailed in the Table.

Within each stratum, non-cases were selected using simple random sampling without replacement. Selection probabilities varied across strata, with near-complete inclusion of individuals with psychiatric hospitalisation and high inclusion of individuals receiving outpatient mental healthcare. Within emergency department, primary care, and other healthcare settings, higher selection probabilities were assigned to individuals with mental disorder indicators, prioritised hierarchically as complex chronic psychiatric patient, chronic psychiatric patient, and depression. This approach was chosen in collaboration with PADRIS to restrict the overall number of sampled individuals for data handling feasibility while retaining detailed exposure information in groups at higher risk of self-harm.

Inverse probability weights

IPWs were assigned to all sampled individuals to account for the disproportionate stratified sampling of non-cases. For cases, the selection probability was 1, and the corresponding weight was 1. For non-cases, weights were calculated as the inverse of the stratum-specific selection probability.

Weights were not stabilised or trimmed. In analyses conducted at the healthcare-contact level, individual-level weights are replicated across all contacts belonging to the same individual.

To facilitate numerical stability and appropriate estimation of uncertainty, IPWs were rescaled such that the sum of the weights equalled the total analytic sample size (n = 789,314). This rescaling does not affect relative weighting between individuals or population-representative inference, but ensures that weighted analyses correspond to the effective sample size of the analytic dataset.

Because selection into the analytic dataset depended only on observed healthcare-setting characteristics and mental disorder indicators, which were accounted for through inverse probability weighting, weighted analyses can be interpreted as representative of the underlying population (i.e., all residents of Catalonia who were alive at any point during the period 2014–2019).

Handling of suicide deaths in the sampling design

Suicide deaths were not used for case definition and as a sampling criterion because mortality data were not yet available at the time of sampling. Hence, suicide deaths could occur among both cases and non-cases. All suicides occurring among individuals classified as cases of suicidal ideation or intentional self-harm were included with selection probability 1 (and weight = 1), whereas suicides occurring among non-cases were included only if the individual was selected through the stratified sampling of non-cases and were assigned the corresponding IPW. As a result, weighted analyses allow population-representative estimation of suicide outcomes, despite the fact that not all suicide deaths in the eligible population were directly sampled. The inclusion of suicide counts by case status and stratum in **Table 1** illustrates this design feature.

Internal validation of the sampling design and weighting approach

As an internal consistency check of the sampling design and weighting approach, the analytic dataset was used to estimate weighted incidence rates of suicide for the period 2014–2019 among individuals aged ≥10 years (645,571 sampled individuals, representing 6,623,221 eligible residents). The estimated suicide incidence rate was 6.8 per 100,000 person-years (95% CI: 5.6–8.0) in the overall population, with sex-specific rates of 3.4 (95% CI: 2.5–4.3) among females and 10.4 (95% CI: 8.1–12.7) among males. These estimates are closely aligned with official suicide mortality statistics reported by the Spanish National Institute of Statistics (INE) [10] for Catalonia, which for the years with available data (2018–2019) report overall rates of 6.95–7.03 per 100,000, with corresponding sex-specific rates of 3.67–3.99 among females and 10.21–10.37 among males. The close agreement between weighted estimates derived from the analytic dataset and official population statistics supports the validity of the sampling design and inverse probability weighting for population-representative analyses.

Supplementary Methods Table 1. Population stratification, sampling, and weighting scheme.

				Cases			Non-Cases					Selected	
Stratum[a]	Healthcare setting	Mental Disorder Indicator	Eligible population	Eligible = Selected	IPW	Suicides[b]	Eligible	Selected	P(sel)	IPW	Suicides among Selected[b]	Total	Suicides[b]
			n	n	1/P(sel)	n	n	n	p	1/P(sel)	n	n	n
1	Psychiatric Hospitalization		54,464	18,546	1.000	271	35,918	35,024	0.975	1.026	156	53,570	427
2	Mental Health Outpatient Visit		465,488	25,067	1.000	168	440,421	193,421	0.439	2.277	162	218,488	330
3	Emergency Department Visit	Complex Chronic Psychiatry Patient[c]	148,263	6,213	1.000	48	142,050	42,014	0.296	3.381	33	48,227	81
4		Chronic Psychiatry Patient[d]	81,776	2,739	1.000	17	79,037	19,970	0.253	3.958	8	22,709	25
5		Depression[e]	488,544	7,742	1.000	49	480,802	55,559	0.116	8.654	20	63,301	69
6		Other	3,935,766	28,721	1.000	87	3,907,045	305,357	0.078	12.795	25	334,078	112
7	General Hospitalization	Complex Chronic Psychiatry Patient[c]	6,029	68	1.000	0	5,961	151	0.025	39.477	2	219	2
8		Chronic Psychiatry Patient[d]	3,280	30	1.000	1	3,250	77	0.024	42.208	0	107	1
9		Depression[e]	28,524	99	1.000	1	28,425	331	0.012	85.876	0	430	1
10		Other	221,186	280	1.000	3	220,906	2,457	0.011	89.909	0	2,737	3
11	Primary Care Visit	Complex Chronic Psychiatry Patient[c]	20,651	372	1.000	12	20,279	629	0.031	32.240	2	1,001	14
12		Chronic Psychiatry Patient[d]	31,688	272	1.000	3	31,416	617	0.020	50.917	2	889	5
13		Depression[e]	143,558	601	1.000	15	142,957	1,790	0.013	79.864	1	2,391	16
14		Other	2,230,845	2,067	1.000	20	2,228,778	30,434	0.014	73.233	4	32,501	24
15	Other		802,093	782	1.000	9	801,351	7,884	0.010	101.643	1	8,666	10
		**Total:**	**8,662,155**	93,599		704	8,568,596	695,715			416	**789,314**	1,120

a. Individuals were assigned to a stratum if the corresponding healthcare setting or mental disorder indicator was recorded at least once in any linked healthcare or registry data during the period 2014–2019, irrespective of timing or frequency. Strata were made mutually exclusive through hierarchical assignment, such that each individual was assigned to the highest applicable stratum (e.g., an individual with both a psychiatric hospitalisation and a primary care visit during 2014–2019 was assigned to the psychiatric hospitalisation stratum).

b. Suicide deaths were not used for sampling. Suicide counts are shown by case status and stratum to illustrate that suicides occurred among both cases and non-cases; population-representative analyses of suicide outcomes require application of IPW.

c. Complex Chronic Psychiatry Patient (Pacient crònic complex en salut mental) refers to a person with a chronic mental disorder who additionally presents high clinical and care complexity, characterised by a combination of severity, comorbidity, functional impairment, and/or intensive or recurrent use of mental health services.

d. Chronic Psychiatry Patient (Pacient crònic en salut mental) refers to a person with a diagnosed mental disorder considered chronic, identified through recorded ICD diagnostic codes in administrative healthcare data, irrespective of current care intensity or service complexity.

e. Depression was defined as the presence of a clinically diagnosed depressive disorder, identified through recorded ICD-9-CM or ICD-10(-CM) diagnostic codes for depressive disorders in administrative healthcare data.

Supplementary Methods Table 2: ICD codes to identify self-harm in routine electronic healthcare data.

	ICD-9-CM	ICD-10-CM	ICD-10
**Intentional self-harm**	**E950*–E958***- Self-poisoning, hanging, strangulation, suffocation, firearms, jumping, others	**T14.91*** - Suicide attempt<br>**T36*–T50* with a 5/6th character of 2** - Drug poisoning (overdose)<br>**T51*–T64*, T65.0*–1*, T65.3*–9* with a 5/6th character of 2** - Toxic effects of nonmedicinal substances<br>**T71* with a 5/6th character of 2** - Asphyxiation, suffocation, strangulation<br>**X71*–X83*** - Drowning and submersion; firearms; explosive or thermal material; sharp or blunt objects; jumping from a high place; jumping or lying in front of a moving object; crashing of a motor vehicle; others	**X60*–X64*** - Drug poisoning (overdose)<br>**X65*–X69*** - Toxic effects of nonmedicinal substances<br>**X70*** - Asphyxiation, suffocation and strangulation<br>**X71*–X84*** - Drowning and submersion; firearms; explosive or thermal material; sharp or blunt objects; jumping from a high place; jumping or lying in front of a moving object; crashing of a motor vehicle, others
**Undetermined self-harm**	**E980*–E987*, E988.1–8** - Self-poisoning, hanging, strangulation, suffocation, firearms, jumping, others	**T36*–T50* with a 5/6th character of 4** - Drug poisoning (overdose)<br>**T51*–T64*, T65.0*–1*, T65.3*–9* with a 5/6th character of 4** - Toxic effects of nonmedicinal substances<br>**T71* with a 5/6th character of 4** - Asphyxiation, suffocation, strangulation<br>**Y21*–Y32*** - Drowning and submersion; firearms; explosive or thermal material; sharp or blunt objects; jumping from a high place; jumping or lying in front of a moving object; crashing of a motor vehicle; others	**Y10*–Y14*** - Drug poisoning (overdose)<br>**Y15*–Y19*** - Toxic effects of nonmedicinal substances<br>**Y20*** - Asphyxiation, suffocation, strangulation<br>**Y21*–Y32*** - Drowning and submersion; firearms; explosive or thermal material; sharp or blunt objects; jumping from a high place; jumping or lying in front of a moving object; crashing of a motor vehicle; others
**Injuries and intoxications possibly related to self-harm**	**881*; 903.2–903.4 and no external cause code is registered**[a] - Open wound of elbow, forearm, and wrist; injury radial/ulnar vessels; injury palmar artery<br>**965*; 967*; 969* and no external cause code is registered**[a] - Poisoning by analgesics,	**S51.00*; S51.80*; S55.0*–S55.1*; S61.5*; S65.0*; S65.1*** - Open wound of elbow / forearm / wrist, injury of ulnar / radial artery at forearm/wrist/arm level<br>**T36*–T50* with 5/6th character missing** - Drug poisoning (overdose)	**S51.8–S51.9, S55.0–S55.1, S61.8–S61.9, S65.0–S65.1** - Open wound of elbow / forearm / wrist, injury of ulnar / radial artery at forearm/wrist/arm level<br>**T39*, T40.0–4, T40.6, T42.3–T42.4, T42.6–T42.7, T43*, T50.7** - Drug poisoning (overdose),

	antipyretics, antirheumatics, sedatives and hypnotics<br>**994.7 and no external cause code is registered[a]** - Asphyxiation / strangulation	**T51*–T64*, T65.0*–1*, T65.3*–9* with 5/6th character missing** - Toxic effects of nonmedicinal substances<br>**T71* with 5/6th character missing -** Asphyxiation, suffocation, strangulation	including non-opioid analgesics and antipyretics, opioids and other narcotics, antiepileptics and sedative-hypnotics, antidepressants, antipsychotics, psychostimulants, other psychotropic drugs, and related substances
**Suicidal ideation**	**V62.84**	**R45.851**	/

(1) Asterisks indicate inclusion of all subcodes.*

(2) Selection of ICD codes for the "Injuries and intoxications possibly related to self-harm" category was based on an extensive MEDLINE search [11-14], including a key overview article providing recommendations on the use of ICD codes for surveillance of self-injurious behaviour [15] (see also [16]).

(3) For selected ICD-9-CM injury and poisoning codes (881; 903.2–903.4; 965*; 967*; 969*; and 994.7), classification of intent required an accompanying external cause (E-) code indicating intentional self-harm, undetermined intent, or accident. When no E-code was recorded, intent could not be ascertained; these records were therefore classified as injuries and intoxications possibly related to self-harm.*

(4) For ICD-10-CM poisoning and injury codes (S- and T-codes), the fifth or sixth character was used to distinguish intentional self-harm, undetermined intent, or absence of specified intent [15]. Records in which this character was missing or did not specify intent were classified as injuries and intoxications possibly related to self-harm.

(5) For ICD-10-CM codes, where applicable, only initial encounters were considered, defined by ICD-10-CM injury and poisoning codes carrying a seventh-character extension of "A", with subsequent encounters ("D") and sequelae ("S") excluded.

(6) ICD-10 codes were used only in <1% of cases and correspond to legacy WHO ICD-10 coding present in a small subset of registry records [17].

References

1. Agència de Qualitat i Avaluació Sanitàries de Catalunya (AQuAS). Programa d'Analítica de Dades per a la Recerca i la Innovació en Salut (PADRIS) [Internet]. Barcelona: AQuAS; [cited 2026 March 5]. Available from: http://aquas.gencat.cat/ca/fem/intelligencia-analitica/padris/
2. Breslow NE, Cain KC. Logistic regression for two-stage case-control data. Biometrika. 1988;75(1):11–20.
3. Chatterjee N, Chen YH, Breslow NE. A pseudoscore estimator for regression problems with two-phase sampling. J Am Stat Assoc. 2003;98(461):158–68.
4. Reilly M. Optimal sampling strategies for two-stage studies. Am J Epidemiol. 1996;143(1):92–100.
5. Robins JM, Rotnitzky A, Zhao LP. Estimation of regression coefficients when some regressors are not always observed. J Am Stat Assoc. 1994;89(427):846–66.
6. Scott AJ, Wild CJ. Fitting logistic regression models in stratified case-control studies. 1991;47(2):497–510.
7. Wimberley T, Plana-Ripoll O, Pedersen CB, Pedersen MG, Dreier JW, Christensen J, et al. Population-representative inference for primary and secondary outcomes in extended case-cohort designs. Discov Public Health. 2025;22(1):599.
8. O'Brien KM, Lawrence KG, Keil AP. The case for case-cohort: an applied epidemiologist's guide to reframing case-cohort studies to improve usability and flexibility. Epidemiology. 2022;33(3):354–61.
9. Pérez V, Elices M, Prat B, Vieta E, Blanch J, Alonso J, et al. The Catalonia Suicide Risk Code: a secondary prevention program for individuals at risk of suicide. J Affect Disord. 2020;268:201–5.
10. Instituto Nacional de Estadística (INE). Tasa de mortalidad por suicidio por comunidad autónoma, edad, sexo y periodo [Internet]. Madrid: INE; [cited 2026 Jan 17]. Available from: https://www.ine.es/jaxi/Tabla.htm?tpx=46688
11. Barak-Corren Y, Castro VM, Javitt S, Hoffnagle AG, Dai Y, Perlis RH, et al. Predicting suicidal behavior from longitudinal electronic health records. Am J Psychiatry. 2017;174(2):154–62.
12. Iribarren C, Sidney S, Jacobs DR, Weisner C. Hospitalization for suicide attempt and completed suicide: epidemiological features in a managed care population. Soc Psychiatry Psychiatr Epidemiol. 2000;35(7):288–96.
13. Walkup JT, Townsend L, Crystal S, Olfson M. A systematic review of validated methods for identifying suicide or suicidal ideation using administrative or claims data. Pharmacoepidemiol Drug Saf. 2012;21 Suppl 1:174–82.
14. Mejías-Martín Y, Martí-García C, Rodríguez-Mejías C, Valencia-Quintero JP, García-Caro MP, Luna J de D. Suicide attempts in Spain according to prehospital healthcare emergency records. PLoS One. 2018;13(4):e0195370.
15. Hedegaard H, Schoenbaum M, Claassen C, Crosby A, Holland K, Proescholdbell S. Issues in developing a surveillance case definition for nonfatal suicide attempt and intentional self-harm using International Classification of Diseases, Tenth Revision, Clinical Modification (ICD-10-CM) coded data. Natl Health Stat Rep. 2018;(108):1–19.

16. Mortier P, Vilagut G, Puértolas Gracia B, De Inés Trujillo A, Alayo Bueno I, Ballester Coma L, et al. Catalonia Suicide Risk Code Epidemiology (CSRC-Epi) study: protocol for a population-representative nested case-control study of suicide attempts in Catalonia, Spain. BMJ Open. 2020;10(7):e037365.

17. Servei Català de la Salut (CatSalut). CIM-10 [Internet]. Barcelona: CatSalut; [cited 2026 Jan 29]. Available from: http://catsalut.gencat.cat/ca/proveidors-professionals/registres-catalegs/catalegs/diagnostics-procediments/cim-10/
