# Supplementary material for: Risk Alert System to Facilitate Universal Suicide Risk Screening in Emergency Care: Protocol for a Prediction Model and Software Development Study Using a Co-Design Approach
Source: JMIR Res Protoc. 2026 Sep 16;15:e100498. doi: 10.2196/100498 (PMC13628038; doi:10.2196/100498)
Supplement: Supplementary file 3 [file resprot_v15i1e100498_app3.pdf]

3 -202220

Project ID: **22**
Title: **Towards Personalized Suicide Prevention - Improving Clinical Risk Assessment and Indicated Treatment Allocation among Patients at Risk for Suicide.**

Summary to the applicants:

The aim of this proposal is the development of a prototype of a Clinical Decision Support System (CDSS), i.e., a medical software program that assists clinicians in the personalized evaluation and management of suicide risk. This seems promising in order to contribute to an improved suicide risk prediction and a more personalised treatment to assess suicide risk. The project will benefit from the use of machine learning techniques based on a range of existing patient data, including the access to key regional data (i.e., Catalan Data Analytics Program for Health Research and Innovation (PADRIS)). This is the main strength of the project, since relevant estimates are based on the entire target population and there can be a direct link with stakeholders. Similarly, the pre-existence of this substantial linked database on which the prototype can be developed removes major potential risks associated with data gathering, linkage and cleaning from a variety of sources.

In addition, the proposal considers not only the technical aspects of development of a suicide risk prediction algorithm, but also how such a tool can be used to improve treatment approaches and outcomes (for patients) and to facilitate clinical decision-making experience for clinicians (i.e., how the tool might affect policy as related to clinical decision-making guidelines and resource allocation).

However, some methodological weaknesses, at least in the description of planned activities, have been detected. The description of the integration of the different data sources is not clear enough: i.e., the use of patients' perspectives in the development of the Clinical Decision Support System. Moreover, contextual factors related to unmet needs are not sufficiently taken into account. Similarly, there are not enough details on the management of risks and barriers associated with the project, e.g., the engagement of clinicians (will they support the project?). And finally, the models will be developed using administrative data and suicide risk of individual patients will be estimated using data entered by the clinician. Are the models appropriate when the input data is different?

The research team appears to have the technical skills and expertise to carry out the proposed work. The combination of a team strong in psychiatric epidemiology and a team strong in machine learning techniques is an excellent combination. However, it is not clear if the team members have experience in mixed methods research. The involvement of researchers with skills and expertise related to health ethics might be useful.

In sum, the project might be appropriate for funding, since it seems to show a positive return considering the health problem addressed and related social costs. This includes a translational support to the clinician-patient encounter, through a personalized approach. Although there is no reference to a potential suicide risk reduction, it seems that this project pursues the engagement of the patient throughout a more perceived care, with a resulting better compliance and treatment adherence.
